# Microbial Diversity Similarities in Periodontal Pockets and Atheromatous Plaques of Cardiovascular Disease Patients

**DOI:** 10.1371/journal.pone.0109761

**Published:** 2014-10-16

**Authors:** Wagner Serra e Silva Filho, Renato C. V. Casarin, Eduardo L. Nicolela Junior, Humberto M. Passos, Antônio W. Sallum, Reginaldo B. Gonçalves

**Affiliations:** 1 Federal University of Piauí, Piauí, Brazil; 2 Paulista University, São Paulo, Brazil; 3 Center of Hemodynamics, Emcor, Piracicaba, Brazil; 4 Piracicaba Dental School, State University of Campinas, São Paulo, Brazil; 5 Groupe de Recherche en Ecologie Bucale, Université Laval, Quebec, Canada; University of Toronto, Canada

## Abstract

**Background and Objective:**

The immune and infectious alterations occurring in periodontitis have been shown to alter the development and severity of cardiovascular disease. One of these relationships is the translocation of oral bacteria to atheroma plaques, thereby promoting plaque development. Thus, the aim of this study was to assess, by 16s cloning and sequencing, the microbial diversity of the subgingival environment and atheroma plaques of patients concomitantly suffering from periodontitis and obstructive coronary artery atherosclerosis (OCAA).

**Methods:**

Subgingival biofilm and coronary balloons used in percutaneous transluminal coronary angioplasty were collected from 18 subjects presenting with generalized moderate to severe periodontitis and OCAA. DNA was extracted and the gene 16S was amplified, cloned and sequenced.

**Results:**

Significant differences in microbial diversity were observed between both environments. While subgingival samples mostly contained the phylum *Firmicutes*, in coronary balloons, *Proteobacteria* (p<0.05) was predominant. In addition, the most commonly detected genera in coronary balloons were *Acinetobacter, Alloprevotella, Pseudomonas, Enterobacter, Sphingomonas* and *Moraxella*, while in subgingival samples *Porphyromonas, Filifactor, Veillonella, Aggregatibacter* and *Treponema* (p<0.05) were found. Interestingly, 17 identical phylotypes were found in atheroma and subgingival samples, indicating possible bacterial translocation between periodontal pockets and coronary arteries.

**Conclusion:**

Periodontal pockets and atheromatous plaques of cardiovascular disease patients can present similarities in the microbial diversity.

## Introduction

Cardiovascular diseases (CVDs), namely coronary heart disease (CHD), stroke, congestive heart failure, and peripheral artery disease, became the leading cause of chronic disease morbidity and mortality in industrialized countries in the twentieth century [1], [2]. It is widely accepted that a major component of pathology in cardiovascular disease (CVD), particularly in atherosclerosis involves multiple components of the innate and adaptive immune systems, leading to an inflammatory response within the atheromatous lesion [3].

In recent decades, oral biofilm, especially in periodontitis patients, has been associated with CVD. Individuals suffering from periodontitis on average present with 14–15% greater risk of developing CVD based on prospective trials, whilst the odds increased to more than 100% when analyzing case–control studies compared to healthy individuals [4]. A recent cohort study report on CVD among 1400 dentate men aged 60–70 years showed that severe loss of periodontal attachment conferred a statistically significant doubled risk of death compared with controls (15.7% versus 7.9%) [5]. The hazard ratio, adjusted for age, smoking, diabetes, hypertension, body mass index, cholesterol, education and marital status and history of a vascular event, was found to be 1.57 (95% confidence interval (CI), 1.04–2.36). These associations have been confirmed in a number of systematic reviews [6]–[9].

Links between periodontitis and atherosclerosis would be predicted based on inflammatory mechanisms initiated by bacteria associated with periodontal lesions, locally or systemically, that then influence the initiation or propagation of the atherosclerotic lesion. Studies on atheroma lesions have focused mainly on the detection of well-recognized putative periodontal bacteria. Moreover, it has not yet been confirmed if atheroma lesions present the same clonal type of bacteria as in the periodontal pocket, which would strongly suggest mouth-to-heart translocation and a relationship between the two diseases.

Thus, in this context, we hypothesized that similar clones would be harvested from subgingival periodontal biofilm in periodontal pockets and atheroma plaques from CVD patients. For this, an open-ended technique, i.e. 16S cloning and sequencing, was applied to assess both microbial profiles, allowing for a comparison between the bacterial populations.

## Materials and Methods

### Study design

This study was delineated as a cohort study, which included subjects presenting periodontal disease concomitantly with obstructive coronary artery atherosclerosis (OCAA), characterized by arterial stenosis greater than or equal to 70% obstruction of the artery channel and evident ischemia.

### Ethics statements

This study was approved by Ethics Committee of Piracicaba Dental School, State University of Campinas (under number 036/2008), and all subjects signed a consent form prior the study begin.

### Sample selection

The OCAA subjects were selected from patients that attended the Cardiac Emergency Room (EMCOR) of Santa Isabel Hospital (Piracicaba, Brazil), between September 2007 and July 2008. During this period, 134 patients, undergoing percutaneous transluminal coronary angioplasty (PTCA - indicated after catheterization examination and diagnosis of OCAA), were screened for periodontal disease. Within this population, whose presenting the following inclusion criteria were selected:

- Presence of generalized moderate to severe chronic periodontitis (at least four teeth with probing depth ≥5 mm), examined by one calibrated examiner (Silva Filho WLS - Intra-class correlation = 0.90);
- At least 10 teeth;
- Plaque index ≥30%;
- Bleeding on probing ≥30%;
- Age between 40 and 80 years old;

Subjects who underwent periodontal treatment during the three months prior to the study or who took antibiotics during the three months prior to the study were excluded. Type-2 diabetes mellitus and osteoporosis were considered as exclusion criteria if HbA1c level was >7% (characterizing a poor-controlled diabetic status) and if osteoporosis were diagnosed as very advanced and not-treated condition (determined by surgeons).

After screening, 18 patients fitted the inclusion criteria and were included in the study. Patients were recruited in the preoperative room at EMCOR. The following parameters were evaluated: probing depth - distance from the gingival margin to the bottom of the pocket (PD), bleeding on probing (BOP) [10], plaque index (PI) [11] and the number of missing teeth.

### Collection of subgingival samples

After screening, intra-oral samples were collected from the deepest portion of the periodontal pockets of each teeth presenting PD>5 mm and bleeding. Subgingival biofilm collection was performed after removal of the supragingival biofilm, and isolation with sterile gauze. Sterile Gracey curettes were used for the removal of biofilm, which were stored in microcentrifuge tubes containing Tris-EDTA solution at −20°C.

### Collection of angioplasty balloon samples

The balloons used in the PTCA procedure were carefully removed and placed in microcentrifuge tubes, containing Tris-EDTA solution, and stored in a −20°C freezer. The collection of the balloons was performed with extreme care to avoid contamination, but without changing the technique’s routine for the PTCA procedure.

### DNA amplification and cloning sequencing

The DNA amplification and cloning followed the steps fully described by Casarin et al. [12] and Meulman et al. [13]. Briefly, the 16S rRNA gene was amplified using a universal primer set (27f and 1492r) and the amplicons were cloned into *Escherichia coli* (TOPO-TA cloning kit, Invitrogen, San Diego, CA, USA). Then, they were cultured in Luria Bertani plates and broth media (LB-Top Agar, Sigma-Aldrich, Buchs, Switzerland). Following vector extraction, the product was purified (QIAprep Miniprep Spin, Qiagen, Quebec, QC, Canada) and sequenced at CHUQ, Centre Hospitalier Universitaire de Québec (Université Laval, Québec, QC, Canada) by Sanger method with 3130XL (Applied Biosystems, Grand Island, NY, USA). After sequencing, a partial sequence of 600 bp was generated, aligned, and a similarity matrix was constructed from the alignments using the method described by Jukes and Cantor [14]. The sequences were aligned with the Clustal X 2.0.12 program [15]. Gaps and unknown bases were eliminated with the Bioedit Program. The phylogenetic tree was constructed by the neighbor-joining method [16]. Bootstrap resampling analysis [17] was used to tree-topologies’ confidence estimation. Sequences were compared using the HOMD database [18], with a level of 98.5% sequence identity as the cut-off (sequences 98% or greater similar were considered to be the same species).

### Data management and statistical analysis

For microbiological data, a variance-stabilizing transformation described by Shchipkova et al. [19] was used, promoting a normal distribution of the data. The proportion (p) of each species in the community of each subject was expressed as *X = sin^−1^ (√p).* This transformed variable X were used to determine the most prevalent phylotypes in atheroma and subgingival biofilm samples, considering its distribution and proportion within individuals. A chi-squared test was used to test for the presence or absence of species and genera in subgingival and coronary samples (SAS Institute Inc. release 9.02, Cary, NC, USA). A 5% level of significance was used for data analyses.

## Results

The mean age of the subjects was 59.5±19.5 years, with 38.9% females and 55.6% smokers. The mean number of remaining teeth was 16.5±4.8 and the mean PD was 4.9±2.1 mm. Three of them were diagnosed as Type-2 diabetes mellitus (presenting HbA1c<7%) and other 3 as osteoporotic (Table 1).

**Table 1:** Demographic data of population.

Data	Value
Age (years±SD)	59.5±19.5
Gender (% females)	38.9%
Smoking (% smokers)	55.6%
Type-2 diabetes mellitus (% positive)*	16.6%
Hypertension (% positive)#	66.7%
Osteoporosis (% positive)	16.6%
Number of remaining teeth (n±SD)	16.5±4.8
Probing depth (mm±SD)	4.9±2.1

*SD- Standard deviation;*

*HbA1c<7%;

# *Arterial Pressure >140/80 mmHg;*

With regard to the microbiological analysis, a total of 68 different species were identified in periodontal pockets and 40 in coronary balloons. Table 2 provides the observed phyla and culture status of phylotypes harvested from both samples. A significant difference in *Firmicutes* and *Proteobacteria* could be seen, with the former more prevalent in periodontal pockets and the latter in coronary balloons (p<0.05– Chi-square test). No difference was seen regarding the presence of cultured/not-yet-cultured phylotypes at the two sites (p>0.05– Chi-Square test). Moreover, it is also demonstrate the number of common phylotypes, i.e., the n of phylotypes presented concomitantly in periodontal pocket and coronary samples in each phylum. It is noticed that *Bacteroidetes* and *Firmicutes* are the phyla presenting higher similarity (6 and 3, respectively). Interestingly, some phyla (*Proteobacteria, Synergistetes* and *GN02*) although presenting species in both environments, did not present anyone common phylotypes. Data of all phylotypes are displayed at Appendix S1.

**Table 2:** Distribution (%total clones and number of common phylotypes) regarding Phylo and Culture Status (%) in periodontal pocket and Coronary balloon.

	Periodontal Pocket	Coronary balloon	Number of common phylotypes
***Actinobacteria***	1 (1.5%)	4 (10.0%)	1
***Bacteroidetes***	20 (29.4%)	7 (17.5%)	6
***Firmicutes*** *	26 (38.2%)	7 (17.5%)	3
***Fusobacteria***	5 (7.4%)	2 (5.0%)	2
***Proteobacteria*** *	10 (14.7%)	18 (45.0%)	0
***Spirochaetes***	4 (5.9%)	1 (2.5%)	1
***Synergistetes***	2 (2.9%)	0 (0.0%)	0
***GN02***	0 (0.0%)	1 (2.5%)	0
**Cultured**	51 (75%)	32 (80%)	
**Not-yet cultured**	17 (25%)	8 (20%)	

*indicate statistically difference (Chi-square test, p<0.05).

**Figure 1:**
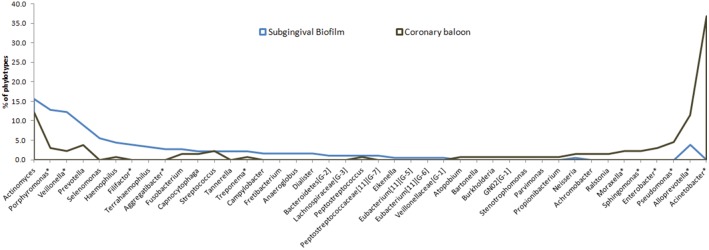
Distribution of clones (% clones) by genus in periodontal pocket and coronary balloon samples. *indicate statistically significant difference between periodontal pocket and coronary balloon (Chi-square test, p<0.05).

Figure 1 shows the distribution of genera in periodontal pockets and coronary balloons (% of total clones). The left panel presents the genera that were statistically highly prevalent at periodontal sites, such as *Porphyromonas, Filifactor, Veillonella, Aggregatibacter* and *Treponema* (p<0.05– Chi-square test). The right panel demonstrates that the most prevalent genera among coronary samples were *Acinetobacter, Alloprevotella, Pseudomonas, Enterobacter, Sphingomonas* and *Moraxella* (p<0.05). Of these, only *Alloprevotella* could also be found in oral samples, and the other genera were exclusive to coronary samples. Other genera, although not statistically significant (p<0.05– Chi-square test), were also exclusively detected in atheroma plaques, for example *GN02 [G-1], Burkholderia, Stenotrophomonas, Parvimonas* and *Propionibacterium* genera.

Table 3 shows the TOP15 phylotypes detected in periodontal pockets and coronary balloons. The phylotypes highly observed in periodontal pockets include most of the well-recognized periodontal pathogens (*Veillonella atypica, Filifactor alocis, Treponema vicentii, Aggregatibacter sp. and Eikenella corrodens,* for example) while the most commonly found species in coronary samples were *Enterobacter hormaechei, Acinetobacter sp., Pseudomonas fluorescens, Acinetobacter baumannii* and *Enterobacter cancerogenus;* these are uncommon species in oral biofilm.

**Table 3:** Top-15 phylotypes (mean of x value±sd and % of positive subjects) detected in periodontal pocket and Coronary balloon.

TOP15 Phylotypes in Periodontal pocket	x value	% of positive subjects
*Actinomyces sp. | HOT-178 | strain B27SC | AF287750 | Unnamed*	*0.35±0.04*	*67%*
*Alloprevotella tannerae | HOT-466 | strain ATCC 51259 | AJ005634 | Named*	*0.18±0.03*	*67%*
*Eikenella corrodens | HOT-577 | strain Moore D25 | GQ422740 | Named*	*0.17±0.07*	*50%*
*Selenomonas sp. | HOT-143 | clone EW051a | AF385497 | Phylotype*	*0.16±0.01*	*67%*
*Streptococcus anginosus | HOT-543 | strain ATCC 33397 | AF104678 | Named*	*0.14±0.05*	*33%*
*Veillonella atypica | HOT-524 | strain DSM 20739 | X84007 | Named*	*0.12±0.03*	*33%*
*Veillonella dispar | HOT-160 | strain DSM 20735 | X84006 | Named*	*0.12±0.02*	*66%*
*Aggregatibacter sp. | HOT-513 | clone MB3_C38 | DQ003635 | Phylotype*	*0.12±0.03*	*33%*
*Fusobacterium naviforme | HOT-689 | strain DMS 20699 | AJ006965 | Named*	*0.10±0.04*	*50%*
*Filifactor alocis | HOT-539 | strain ATCC 35896 | AJ006962 | Named*	*0.10±0.03*	*67%*
*Selenomonas infelix | HOT-639 | strain ATCC 43532 | AF287802 | Named*	*0.09±0.05*	*83%*
*Campylobacter concisus | HOT-575 | strain FDC 288 | L06977 | Named*	*0.09±0.01*	*50%*
*Treponema sp. | HOT-270 | clone DD012 | GQ422733 | Phylotype*	*0.09±0.02*	*33%*
*Anaeroglobus geminatus | HOT-121 | clone BB166 | AF287783 | Named*	*0.08±0.03*	*33%*
*Treponema vincentii | HOT-029 | strain ATCC 35580 | AF033309 | Named*	*0.08±0.03*	*33%*
**TOP15 Phylotypes in Coronary Balloon**		
*Actinomyces sp. | HOT-178 | strain B27SC | AF287750 | Unnamed*	*0.21±0.08*	*50%*
*Enterobacter hormaechei | HOT-634 | strain DSMZ 16691 | AJ853890 | Named*	*0.26±0.25*	*50%*
*Alloprevotella tannerae | HOT-466 | strain ATCC 51259 | AJ005634 | Named*	*0.22±0.11*	*67%*
*Acinetobacter sp. | HOT-408 | clone C4AKM094 | AY278636 | Phylotype*	*0.21±0.05*	*83%*
*Pseudomonas fluorescens | HOT-612 | strain DSM 50090 | Z76662 | Named*	*0.12±0.03*	*67%*
*Acinetobacter baumannii | HOT-554 | strain DSM 30007 | X81660 | Named*	*0.10±0.02*	*83%*
*Enterobacter cancerogenus | HOT-565 | strain LMG 2693 | Z96078 | Named*	*0.10±0.05*	*50%*
*Ralstonia sp. | HOT-027 | strain C37KA | AY005039 | Unnamed*	*0.10±0.04*	*33%*
*Moraxella osloensis | HOT-711 | strain Ben 58 | X95304 | Named*	*0.08±0.10*	*67%*
*Actinomyces sp. | HOT-525 | clone MB6_C03 | DQ003632 | Phylotype*	*0.08±0.02*	*33%*
*Capnocytophaga leadbetteri | HOT-329 | clone BR085 | GU350453 | Named*	*0.08±0.10*	*67%*
*Achromobacter xylosoxidans | HOT-343 | strain DSM 30026 | Y14907 | Named*	*0.08±0.01*	*33%*
*Porphyromonas gingivalis | HOT-619 | strain DSM 20709 | X73964 | Named*	*0.07±0.40*	*67%*
*Prevotella sp. | HOT-472 | clone GU027 | AY349398 | Unnamed*	*0.07±0.29*	*33%*
*Pseudomonas pseudoalcaligenes | HOT-740 | strain LMG 1225 | Z76666 | Named*	*0.07±0.05*	*33%*

**Figure 2:**
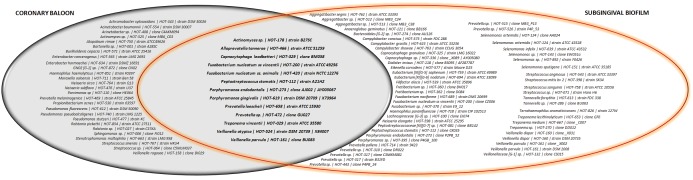
Phylotypes detected in the subgingival biofilm of periodontal pockets (right) and coronary balloons (left). The phylotypes in the center are those detected in both samples within the same individual.

Moreover, in Figure 2, it is possible to identify some clones that were found in both periodontal and coronary balloon samples. Some of these phylotypes are well-recognized periodontal pathogens, such as *P. gingivalis, Treponema vincentii, Fusobacterium nucleatum ss. animalis, Porphyromonas endodontalis* and *Veillonella parvula* and *V. atypica,* but other species, usually described as comensal oral microorganisms, were also detected in atheroma plaques (*Actinomyces sp., Alloprevotella tannerae, Capnocytophaga leadbetteri, Prevotella loescheii, Peptostreptococcus stomatis).* All individuals presented at least one common phylotype in periodontal pocket and coronary balloon samples.

## Discussion

Several studies have shown an increased mortality rate due to CVD in individuals with a higher level of tooth attachment loss [20], [21], which has been correlated to the bacterial load of the oral environment and a consequent immunoinflammatory response against them. This idea has been so important that some papers suggest that periodontal treatment could represent a novel therapeutic opportunity as to tackle the CVD burden and CVD mortality in the general population [21]. To some extent, a recent study seems to corroborate this idea. Amar et al. [22] observed that a *Proteobacteria* and *Eubacteria* load in the plasma of a population monitored over a long period was directly correlated to CVD occurrence, indicating the bacteria could has some role on CVD development.

The way in which the bacteria could impact on CVD has been partially explained. Atheroma lesions may be initiated by inflammatory stimuli, including systemic and locally produced inflammatory cytokines and chemotactic agents that cause changes in the endothelium such as up-regulation of adhesion molecules. These changes promote interactions with leukocytes, such as monocytes, that promote leukocyte migration into the intimal layer of the artery. Activation of the endothelium additionally leads to the release of chemotactic cytokines, such as monocyte chemotactic protein-1 (MCP-1), that further attract monocytes or other cells that can transport bacteria into the lesion. Once inside endothelial layers, bacteria can initiate or exacerbate the host-cell response. For example, *P. gingivalis* induces monocyte migration and can significantly enhance the production of pro-inflammatory cytokines [23]. *P. gingivalis* also induces pro-coagulant effects in human aortic endothelial cells [24], initiates apoptosis and increases mononuclear cell adhesion to endothelial cells [25]. It is well-known that the disease is characterized by the accumulation of cholesterol and recruitment of macrophages into the arterial wall [26]. It can thus be considered both a metabolic and an inflammatory disease [25].

In addition, some studies have attempted to track the origin of bacteria found in atheroma plaques. Direct infection of the endothelium resulting in dysfunction, inflammation and atherosclerosis was first supported by the detection of *Chlamydia pneumonia* in atherosclerotic plaques [27]. Several studies using targeted techniques were able to identify oral microorganisms, especially red complex pathogens, in coronary samples. *T. forsythia* was found in 3.7–61.9% of samples [28]–[31]. Other studies identified *P. gingivalis* in atheromatous plaques, either alone [32] or in concomitant colonization with *P. intermedia*, *T. forsythia* and *A. actinomycetemcomitans* [29], [33], in 4.17 to 85% of samples [34], [35]. *A. actinomycetemcomitans*, another important pathogen, was also identified in coronary samples (5.0–66.7%) [36], [37]. However, it is important to highlight that some studies did not find any oral pathogens in coronary samples [38]–[41]. An important point to be discussed is that, although oral pathogens were not observed, the tests indicated the presence of bacterial DNA in atheroma/coronary samples [39], [41]. This can indicate two important aspects: 1- the *oral microbiota is not the only source of pathogens*; 2- *not only well-recognized oral/periodontal pathogens can colonize plaques and be related to atherosclerotic disease*.

With respect to the bacterial source, studies have attempted to correlate not only oral/periodontal but also gut flora to coronary bacterial diversity. This is important since both locations are major sources of bacteria in transient bacteremia and could be responsible for intra-individual translocation. Koren et al. [42] compared the biodiversity of fecal swabs, oral swabs and specimens obtained during endarterectomy from CVD patients. Using a pyrosequencing analysis, the authors observed that the oral as well as gut microbiome could represent sources of bacteria found in atheroma plaques. Interestingly, they also related oral/gut flora to systemic biomarkers with CVD (especially plasma cholesterol), which could indicate that the oral and/or fecal bacterial community composition can predict and contribute to atherosclerosis development and/or progression. The authors also highlighted that the impact of infection on atherosclerosis is related to the total “pathogen burden,” i.e., the aggregate number of pathogens infecting an individual [42], [43]. Thus, all sources of bacteria should be considered when atherosclerotic lesions are studied.

Recently, Calandrini et al. [44] evaluated 35 endarterectomy specimens using 16S cloning and sequencing, and some of these results corroborate our evidence. The results indicate a higher prevalence of the phylum *Proteobacteria* (78.3% of identified taxa), whereas *Firmicutes* comprised only 21.7% of the identified taxa. In our results, *Proteobacteria* phylum was also the most detectable in coronary samples (45%), and was statistically more prevalent than in subgingival biofilm (14.7%), in which *Firmicutes* was the most prevalent (38.2% and 17.5% in periodontal and coronary pockets, respectively).

Moreover, in this study [44], the family P*seudomonadaceae* was represented by four phylotypes – within them, *Pseudomonas fluorescences.* In our study, *Pseudomonas* genus (belonging to *Gammaproteobacteria* class) was also statistically more prevalent in coronary samples, including, within their phylotypes, *Pseudomonas pseudoalcaligenes | HOT-740 | strain LMG 1225* and *Pseudomonas fluorescens | HOT-612 | strain DSM 50090* (which were two of the TOP15 phylotypes present in coronary samples) as well as *Pseudomonas stutzeri | HOT-477 | strain KC*. Interestingly, no *Pseudomonas* were found in subgingival biofilms, but were exclusively detected in coronary samples, which corroborates a study by Koren et al. [42] in which remarkable levels of *Pseudomonas*, such as *P. luteola*, were reported in atherosclerotic plaques, despite its absence in gut or oral samples [42]. *P. fluorescences* has been previously associated with mechanical ventilator-induced pneumonia [45], but there are no reports of this species in atherosclerotic plaques.

However, in our results, within the *Gammaproteobacteria* class, the most detected genus was *Acinetobacter,* also exclusively detected in coronary samples. Moreover, within this genus, *Acinetobacter sp. | HOT-408 | clone C4AKM094* and *Acinetobacter baumannii | HOT-554 | strain DSM 30007* were the most prevalent phylotypes (found in 50% and 75% of individuals). Previous studies did not identify this genus in coronary samples, but other publications have shown that *Acinetobacter* is of growing interest due to its increased incidence of multidrug resistance (MDR) [46] and its presence in nosocomial infections (63% of cases in the USA [47]). Moreover, it is related to septicemia, pneumonia and death [48]–[50], mostly in immunocompromised patients hospitalized in intensive care units [51].

Other species found exclusively in atheroma specimens should be highlighted. The *Enterobacter* genus was the statistically most prevalent in coronary samples, but not in biofilm. Moreover, two of the TOP15 most detected phylotypes belonging to this genus – *Enterobacter hormaechei | HOT-634 | strain DSMZ 16691* and *Enterobacter cancerogenus | HOT-565 | strain LMG 2693*. *Enterobacter spp.* are opportunistic pathogens that can cause bacteremia and have been associated with soft tissue and systemic infections, including endocarditis and meningitis [52], [53]. Moreover, their resistance to chemotherapeutic drugs [52] has also brought light to this genus, and more attention should be given to its presence in atheroma plaques.

Another genus exclusively detected in coronary samples was *Moraxella spp*. The genus *Moraxella* consists of aerobic, oxidase-positive and Gram-negative coccobacilli. One species of this genus that was detected in 40% of the individuals was *Moraxella osloensis*, which has been isolated from environmental sources in hospitals and from the normal human respiratory tract [54], as well as in bacteremia, central venous catheter-related infection, pneumonia [55] and meningitis [56]. This genus is quite rarely detected in oral samples [57], [58], so an improbable relationship between oral infection and atheromas could be drawn. In any case, it is another pathogen to be considered in CVD and atheroma etiopathogenesis. In summary, it is clear that not only well-recognized oral pathogens are harbored in atheroma plaques and, after the analysis of this and previous studies, more attention should be paid to other phylotypes and their roles in atherosclerotic disease.

Therefore, another interesting result of this study is the fact of some clones could be harvested from subgingival biofilm and atheroma samples within the same individual, confirming the possibility of intra-individual translocation. Thirteen phylotypes were concomitantly detected in oral and coronary disease, some of them well-recognized pathogens, and some previously described in atheroma plaques, such as *P. gingivalis, Fusobacterium nucleatum ss vincentii, Fusobacterium nucleatum ss. animalis, Porphyromonas endodontalis, Treponema vincentii* and *Veillonella parvula*. Moreover, other clones not usually associated to periodontal disease (*Actinomyces sp. | HOT-178 | strain B27SC, Alloprevotella tannerae, Capnocytophaga leadbetteri, Peptostreptococcus stomatis, Prevotella loescheii, Prevotella sp. | HOT-472 | clone GU027* and *Veillonella atypical)* were also detected in the heart and periodontal pockets. Thus, two conclusions can be drawn: 1. *bacterial profile from periodontal pockets and atheroma plaques presented similar microbiota, what could indicate a possibility of translocation, once same clones were detected in both sites;* and 2. *not only periodontal pathogens can translocate from the mouth to the heart* (suggesting that new studies, focusing on different pathogens and not only on periodontal pathogens, should be done).

The relationship between CVD and periodontal disease has been growing in evidence and importance [59]. The present study brings more knowledge to this field; however, more studies should be done. Calandrini et al. [44] made an important statement: “detection of certain bacteria in atherosclerotic plaques does not necessarily mean that these species promote the atherosclerosis”. To clarify this relationship, studies trying to understand how, and if, each pathogen can modulate atherosclerotic disease should be done. So, with this further confirmation, a focus on periodontal health with the intention to reduce the bacterial load and, consequently, the possibility of intra-individual translocation must be considered by physicians, dentists and patients. Moreover, further studies could give more strength evidence of translocation, including, for example, periodontally-healthy individuals. Additionally, should also focus in other than only well-recognized pathogens in view of a microbial diversity infecting atheroma plaque. Another important analysis is the impact of smoking on this relationship between similarities of subgingival and coronary samples. Smoking is a risk factor for both conditions (cardiac disease and periodontitis) and has shown to modulate subgingival microbiota [19]. However, no information regarding the impact of smoking on atheroma biodiversity is disposable. Although the present study included some smokers, this was not a variable considered for data analysis (in view of limited number of subjects). So, further analysis also could be done with this intention.

## Supporting Information

Appendix S1 (DOCX)
