# Supplementary material for: Microbial Diversity Similarities in Periodontal Pockets and Atheromatous Plaques of Cardiovascular Disease Patients
Source: PLoS One. 2014 Oct 16;9(10):e109761. doi: 10.1371/journal.pone.0109761 (PMC4199612; doi:10.1371/journal.pone.0109761)
Supplement: Appendix S1 — (DOCX) [file pone.0109761.s001.docx]

**Appendix S1.** Description and sequences of phylotypes harvested from periodontal pockets and coronary balloon, according to Phylum, Class, Order, Family and Genus.

| ***Periodontal Pocket*** | | | | | | | |
| --- | --- | --- | --- | --- | --- | --- | --- |
| **Phylotype** | | **Sequence** | **Phylum** | **Class** | **Order** | **Family** | **Genus** |
| *Actinomyces sp. \| HOT-178 \| strain B27SC \| AF287750 \| Unnamed* | | CGGCGGGGCCGANCCNAGGGCCTCACTAAACCATCCAATCGGTAGTAGCGACGGGCGGTGTGTACAAAGGGCAGGGACTTAATCAACGCAAGCTTATGACCCGCACTTACTGGGAATTCCTCGTTCATGGGGAANAATTGCAATCCCCGATCCCCATCACGAATGGGGTTCAACGGGTTACCCGCGCCTGCCGGCGTAGGGTAGGCACACGCTGAGCCAGGATCAAACTCTAAGGGCGAATTCCAGCACACTGGCGGCCGTTACTAGTGGATCCGAGCTCGGTACCAAGCTTGGCGTAATCATGGTCATAGCTGTTTCCTGTGTGAAATTGTTATCCGCTCACAATTCCACACAACATACGAGCCGGAAGCATAAAGTGTAAAGCCTGGGGTGCCTAATGAGTGAGCTAACTCACATTAATTGCGTTGCGCTCACTGCCCGCTTTCCAGTCGGGAAACCTGTCGTGCCAGCTGCATTAATGAATCGGCCAACGCGCGGGGAGAGGCGGTTTGCGTATTGGGCGCTCTTCCGCTTCCTCGCTCACTGACTCGCTGCGCTCGGTCGTTCGGCTGCGGCGAGCGGNATCAGCTCACTCAAAGG | *Actinobacteria* | *Actinobacteria* | *Actinomycetales* | *Actinomycetaceae* | *Actinomyces* |
| *Alloprevotella tannerae \| HOT-466 \| strain ATCC 51259 \| AJ005634 \| Named* | | TTTACGGTTACCTTGTTACGACTTAGCCCCAATCACCAGTTTTGCCCTAGGCCGATCCTTGCGGTCACGGACTTCAGGCACCCCCGGCTTTCATGGCTTGACGGGCGGTGTGTACAAGGCCCGGGAACGTATTCACCGCGCCATGGCTGATGCGCGATTACTAGCGAATCCGGCTTCGTGGAGTCGGGTTGCAGACTCCAGTCCGAACTGAGGCCGGGTTTCGGGATCAGCATCCTGTCGCC AGGTAGCTTCCTGCTGCTCCGACCATTGTAACACGTGTGTAGCCCCGGACGTAAGGGCCGTGCTGATTTGACGTCATCCCCACCTTCCTCCGCCTTGCGGCGGCAGTATCCACGGAGTTCCCGGCATTACCCGATGGCAAACGTAGAAAAGGGTTGCGCTCGTTATGGCACTTAAGCCGACACCTCACGGCACGAGCTGACGACAACCATGCAGCACCTCCAAAAGTGTCCGAAGAAAAAGCGCATCTCTGCACTCCGCACTCTTGGTTCAAGCCCGGGTAAGGTTCCTCGCGTATCATCGAATTAAACCACATGTTCCTCCGCTTGTGCGGGCCCCCGTCAATTCCTTTGAGTTTCA | *Bacteroidetes* | *Bacteroides* | *Bacteroidales* | *Prevotellaceae* | *Alloprevotella* |
| *Aggregatibacter segnis \| HOT-762 \| strain ATCC 33393 \| M75043 \| Named* | | TTAGAGTTTGATCATGGCTCAGATTGAACGCTGGCGGCAGGCTTAACACATGCAAGTCGAACGGTAACAGGAGAAAGCTTGCTTTCTTGCTGACGAGTGGCGGACGGGTGAGTAATGCTTGGGAATCTGGCTTATGGAGGGGGATAACGACGGGAAACTGTCGCTAATACCGCGTAGAGTCGAGAGATGAAAGTGCGGGACCGCAAGGCCGCATGCCATAGGATGAGCCCAAGTGGGATTAGGTAGTTGGTGGGGTAATGGCCTACCAAGCCGACGATCTCTAGCTGGTCTGAGAGGATGACCAGCCACACCGGGACTGAGACACGGCCCGGACTCCTACGGGAGGCAGCAGTGGGGAATATTGCGCAATGGGGGCAACCCTGACGCAGCCATGCCGCGTGAATGAAGAAGGCCTTCGGGTTGTAAAGTTCTTTCGGTGACGAGGAAGGCGTGATGTTTAATAGGCATCACGATTGACGTTAATCACAGAAGAAGCACCGGCTAACTCCGTGCCAGCAGCCGCGGTAATACGGAGGGTGCGAGCGTTAATCGGAATAACTGGGCGTAAAGGGCACGCAGGCGGCTATTTAAGTGAGGTGT | *Proteobacteria* | *Gammaproteobacteria* | *Pasteurellales* | *Pasteurellaceae* | *Aggregatibacter* |
| *Aggregatibacter sp. \| HOT-512 \| clone MB3_C24 \| DQ003621 \| Unnamed* | | TTAGGAGTTTGTCATGGCTCAGATTGAACGCTGGCGGCAGGCTTAACACATGCAAGTCGAACGGTAACAGGAGAAAGCTTGCTTTCTTGCTGACGAGTGGCGGACGGGTGAGTAATGCTTGGGAATCTGGCTTATGGAGGGGGATAACGACGGGAAACTGTCGCTAATACCGCGTAGTGTCGAGAGATGAAAGTGCGGGACCGCAAGGCCGCATGCCATAGGATGAGCCCAAGTGGGATTAGGTAGTTGGTGGGGTAAAGGCCTACCAAGCCGACGATCTCTAGCTGGTCTGAGAGGATGACCAGCCACACCGGGACTGAGACACGGCCCGGACTCCTACGGGAGGCAGCAGTGGGGAATATTGCGCAATGGGGGCAACCCTGACGCAGCCATGCCGCGTGAATGAAGAAGGCCTTCGGGTTGTAAAGTTCTTTCGGTGACGAGGAAGGCGTGATGTTTAATAGGCATCACGATTGACGTTAATCACAGAAGAAGCACCGGCTAACTCCGTGCCAGCAGCCGCGGTAATACGGAGGGTGCGAGCGTTAATCGGAATAACTGGGCGTAAAGGGCACGCAGGCGGCTATTTAAGTGAGGTGT | *Proteobacteria* | *Gammaproteobacteria* | *Pasteurellales* | *Pasteurellaceae* | *Aggregatibacter* |
| *Aggregatibacter sp. \| HOT-513 \| clone MB3_C38 \| DQ003635 \| Phylotype* | | TTAGAGTTTGATCATGGCTCAGATTGAACGCTGGCGGCAGGCTTAACACATGCAAGTCGAACGGTAACAGGAGAAAGCTTGCTTTCTTGCTGACGAGTGGCGGACGGGTGAGTAATGCTTGGGAATCTGGCTTATGGAGGGGGATAACGACGGGAAACTGTCGCTAATACCGCGTAGAGTCGAGAGACGAAAGTGCGGGACCGCAAGGCCGCATGCCATAGGATGAGCCCAAGTGGGATTAGGTAGTTGGTGGGGTAATGGCCTACCAAGCTGACGATCTCTAGCTGGTCTGAGAGGATGACCAGCCACACCGGGACTGAGAGACGGCCCGGACTCCTACGGGAGGCAGCAGTGGGGAATATTGCGCAATGGGGGCAACCCTGACGCAGCCATGCCGCGTGAATGAAGAAGGCCT TCGGGTTGTAAAGTTCTTTCGGTATTGAGGAAGGTTGTTGTGTTAATAGCACGACAAATTGACGTTAAATACAGAAGAAGCACCGGCTAACTCCGTGCCAGCAGCCGCGGTAATACGGAGGGTGCGAGCGTTAATCGGAATAACTGGGCGTAAAGGGCACGCAGGCGGACTTTTAAGTGAGGTGT | *Proteobacteria* | *Gammaproteobacteria* | *Pasteurellales* | *Pasteurellaceae* | *Aggregatibacter* |
| *Anaeroglobus geminatus \| HOT-121 \| clone BB166 \| AF287783 \| Named* | | TTAGAGTTTGATCCTGGCTCAGGACGAACGCTGGCGGCGTGCGTAACACATGCAAGTCGAACGAGAGAGTGAGAGAAGCTTGCTTTTCTTACGATCGAGTGGCAAACGGGTGAGTAACGCGTAAACAACCTGCCCCGCAGATGGGGACAACAGCTGGAAACGGCTGCTAATACCGAATACGGTCCTCTTAGCGCATGGTAAGAGGAAGAAAGGGTGGCCTCTGGAACAAGCTACCGCTGCGGGAGGGGTTTGCGTCTGATTAGCTGGTTGGAGGGGTAACGGCCCACCAAGGCGACGATCAGTAGCCGGTCTGAGAGGATGAACGGCCACATTGGAACTGAGACACGGTCCAGACTCCTACGGGAGGCAGCAGTGGGGAATCTTCCGCAATGGGCGAAAGCCTGACGGAGCAACGCCGCGTGAGTGAAGACGGCCTTCGGGTTGTAAAGCTCTGTTATAGGGGACGAACGGCCGGGTAGCGAAGAGGTAGCCGGCATGACGGTACCGTAAGAGAAAGCCACGGCTAACTACGTGCCAGCAGCCGCGGTAATACGTAGGTGGCAAGCGTTGTCCGGAATGATTGGGCGTAAAGGGCGCGCA | *Firmicutes* | *Clostridia* | *Clostridiales* | *Veillonellaceae* | *Anaeroglobus* |
| *Bacteroidales [G-2] sp. \| HOT-274 \| clone AU126 \| AY005072 \| Unnamed* | | TTTACGGCTACCTTGTTACGACTTAGCCCCAGTCATCGGTTTTACCCTAGGTCGCTCCTTGCGGTCACGAACTTCAGGTCCCCCCAACTCCCATGGCTTGACGGGCGGTGTGTACAAGGCCCGGGAACGTATTCACCGCGCCATGGCTGATGCGCGATTACTAGCGAATCCAGCTTCACGGAGTCGAGTTGCAGACTCCGATCCGAACTGAGAAAAGTTTTAAGGGATTAGCATCACCTCGCGGTGTAGCAACCCTCTGTACTCCCCATTGTAACACGTGTGTCGCCCCGGACGTAAGGGCCGTGCTGATTTGACGTCATCCCCACCTTCCTCACACCTTACGGTGGCAGTCCCAATAGAGTCCCCATCTGAATGCTGGTAACTATTGGCAAGGGTTGCGCTCGTTATGGCACTTAAGCCGACACCTCACGGCACGAGCTGACGACAACCATGCAGCACCTACACTATCGCCATTGCTGGATAACGAATCTCTCCGTCGGTCGATAGCATTTCAAGCCCGGGTAAGGTTCCTCGCGTATCATCGAATTAAACCACATGTTCCTCCGCTTGTGCGGGCCCCCGTCAATTCCTTTGAGTTT | *Bacteroidetes* | *Bacteroides* | *Bacteroidales* | *Bacteroidetes[F-2]* | *Bacteroidetes[G-2]* |
| *Campylobacter concisus \| HOT-575 \| strain FDC 288 \| L06977 \| Named* | | TTTACGGTTACCTTGTTACGACTTCACCCCAGTCGCTGATTCCACTGTGGACGGTAACTAATTTAGTATTCCGGCTTCGAGTGAAATCAACTCCCATGGTGTGACGGGCGGTGAGTACAAGACCCGGGAACGTATTCACCGTAGCATGGCTGATCTACGATTACTAGC GATTCCGGCTTCATGGAGTCGAGTTGCAGACTCCAATCCGAACTGGGACATATTTTATAGATTTGCTCCATCTCGCGATATTGCTTCTCATTGTATATGCCATTGTAGCACGTGTGTCGCCCCGGACATAAGGGCCATGATGACTTGACGTCGTCCACACCTTCCTCCTCCTTACGAAGGCAGTCTCATTAGAGTGCTCAGCCGAACTGTTAGCAACTAATGACGTGGGTTGCGCTCGTT GCGGGACTTAACCCAACATCTCACGACACGAGCTGACGACAGCCGTGCAGCACCTGTCTTAACATTTCTGCAAGCAGACACTCTTCTATCTCTAGATGATTTGTTAGATATCAAGTCCGGGTAAGGTTCTTCGCGTATCTTCGAATTAAACCACATGCTCCACCGCTTGTGCGGGTCCCCGTCTATTCCTTT | *Proteobacteria* | *Epsilonproteobacteria* | *Campylobacterales* | *Campylobacteraceae* | *Campylobacter* |
| *Campylobacter gracilis \| HOT-623 \| strain ATCC 33236 \| L04320 \| Named* | | TTAGAGTTTGATCATGGCTCAGAGTGAACGCTGGCGGCGTGCCTAATACATGCAAGTCGAACGGAATTTAAAAGAGCTTGCTCTTTTAAATTTAGTGGCGCACGGGTGAGTAATATATAGCTAATCTGCCCCTTGCTGGAGGACAACAGTTAGAAATGACTGCTAATACTCCATACTCCTTTTTACCATAAGATAAATCGGGAAAGAATTTCGGCAAGGGATGAGACTATATCGTATCAGCTAGTCGGTGGGGTAACGGCCTACCGAGGCTATGACGCGTAACTGGTCTGAGAGGATGATCAGTCACATTGGAACTGAGACACGGTCCAAACTCCTACGGGAGGCAGCAGTAGGGAATATTGCGCAATGGGGGAAACCCTGACGCAGCAACGCCGCGTGGAGGATGACACTTTTCGGAGCGTAAACTCCTTTTGTTAGGGAAGAATAATGACGGTACCTAACGAATAAGCACCGGCTAAC TCCGTGCCAGCAGCCGCGGTAATACGGAGGGTGCAAGCGTTACTCGGAATCACTGGGCGTAAAGGACGCGTAGGCGGATTATCAAGTCTCTTGTGAAATCTAACGGCTCAACCGTTAAAC | *Proteobacteria* | *Epsilonproteobacteria* | *Campylobacterales* | *Campylobacteraceae* | *Campylobacter* |
| *Campylobacter showae \| HOT-763 \| strain CCUG 3054 \| L06974 \| Named* | | TTAGAGTTTGATCATGGCTCAGAGTGAACGCTGGCGGCGTGCCTAATACATGCAAGTCGAACGGAGATTAAGTAGCTTGCTATTTAATCTTAGTGGCGCACGGGTGAGTAATATATAGCTAACTTGCCCATTACTAAGGGACAACAGTTGGAAACGACTGCTAATACCTTATACTCCGTATCTATATAAGTAGATACGGGAAAGTTTTTCGGTAATGGATAGGGCTATATCGTATCAGCTAGTTGGTAAGGTAATGGCTTACCAAGGCTATGACGCGTAACTGGTCTGAGAGGATGATCAGTCACACTGGAACTGAGACACGGTCCAGACTCCTACGGGAGGCAGCAGTAGGGAATATTGCTCAATGGGGGAAACCCTGAAGCAGCAACGCCGCGTGGAGGATGACACTTTTCGGAGCGTAAACTCCTTTTCTTGGGAAAGAATTATGACGGTACCCAAGGAATAAGCACCGGCTAACTCCGTGCCAGCAGCCGCGGTAATACGGAGGGTGCAAGCGTTACTCGGAATCACTGGGCGTAAAGGACGCGTAGGCGGATTATCAAGTCTCTTGTGAAATCTAACGGCTTAACCGTTAAACTG | *Proteobacteria* | *Epsilonproteobacteria* | *Campylobacterales* | *Campylobacteraceae* | *Campylobacter* |
| *Capnocytophaga granulosa \| HOT-325 \| strain LMG 16022 \| U41347 \| Named* | | TTAGAGTTTGATCATGGCTCAGGATGAACGCTAGCGGCAGGCCTAACACATGCAAGTCGAGGGAGAAGCCCTTCGGGCAGAAACCGGCGCACGGGTGCGTAACGCGTATGCAACCTACCTTTCACAGGGGGATAGCCCGAAGAAATTTGGATTAATACCCCATAATATTATTGGATGGCATCATTTGATAATTAAAATTACGATGGTAAAAGATGGGCATGCGTCCTATTAGCTAGTTGGAGTGGTAACGGCACCCCAAGGCTACGATAGGTAGGGGTCCTGAGAGGGAGATCCCCCACACTGGTACTGAGACAGGGACCAGACTCCTACGGGAGGCAGCAGTGAGGAATATTGGTCAATGGTCGGAAGACTGAACCAGCCATGCCGCGTGCAGGAAGAATGCCTTATGGGTTGTAAACTGCTTTTATATGGGAAGAATAAGGAGTACGTGTACTTTGATGACGGTACCATATGAATAAGCATCGGCTAACTCCGTGCCAGCAGCCGCGGTAATACGGAGGATGCGAGCGTTATTCGGAATCATTGGGTTTAAAGGGTCTGTAGGCGGGCTATTAAGTCAGGGGTGAAAGGTTTCAGCTT | *Bacteroidetes* | *Flavobacteria* | *Flavobacteriales* | *Flavobacteriaceae* | *Capnocytophaga* |
| *Capnocytophaga leadbetteri \| HOT-329 \| clone BM058 \| AY005075 \| Named* | | TTTACGGTTACCTTGTTACGACTTAGCCCCAGTCACTAGTTTTACCCTAAACAGCTCCTCGCGGTGACCGTCTTCAGGTACCCCCAGCTTCCATGGCTTGACGGGCGGTGTGTACAAGGCCCGGGAACGTATTCACCGGATCATGGCTGATATCCGATTACTAGCGATTCCAGCTTCACGGAGTNCGAGTTGCAGACTCCGATCCGAACTGTGATCGTCTTTATAGATTCGCGCCTGCTCACGCAGTGGCTGCTCTCTGTAACGACCATTGTAGCACGTGTGTAGCCCAAGATGTAAGGGCCGTGATGATTTGACGTCATCCCCACCTTCCTCACGGTTTGCACCGGCAGTCCCACTAGAGTGCTCGACTCGACTCGCTAGCAACTAATGGCAGGGGTTGCGCTCGTTATAGGACTTAACCTGACACCTCACGGCACGAGCTGACGACAACCATGCAGCACCTTGAAAACTGTCCGAAGAAAANCGTATCTCNACACCTGTCNNTCTCCATTTAAACCTTGGTAANNNTNCTCGCGTATCANCNAATTAAANCACATGCTCCANCGCTTGTGCGGGCCCCCNTCNATTCCTTTGAGTTTC | *Bacteroidetes* | *Flavobacteria* | *Flavobacteriales* | *Flavobacteriaceae* | *Capnocytophaga* |
| *Capnocytophaga sp. \| HOT-336 \| clone _X089 \| AY005080 \| Unnamed* | | TTTACGGCTACCTTGTTACGACTTAGCCCCAGTCACTAGTTTTACCCTAAACAGCTCCTTTAGGTAACCGTCTTCAGGTACTCCCAGCTTCCATGGCTTGACGGGCGGTGTGTACAAGGCCCGGGAACGTATTCACCGGATCATGGCTGATATCCGATTACTAGCGATTCCAGCTTCACGGAGTCGAGTTGCAGACTCCGATCCGAACTGTGACCGTCTTTATAGATTCGCGCCTGCTCACGCAGTGGCTGCTCTCTGTAACGGCCACTGTAGCACGTGTGTAGCCCAAGATGTAAGGG CCGTGATGATTTGACGTCATCCCCACCTTCCTCTCGGTTTGCACCGGCAGTCCCATTAGAGGGCTCGACTCGACTCGTTAGCAACTAATGGCAGGGGTTGCGCTCGTTATAGGACTTAACCTGACACCTCACGGCACGAGCTGACGACAACCATGCAGCACCTTGAAAACTGTCCGAAGAAAAAGGTATCTCTACCTCTGTCAGTCCCCATTTAAACCTTGGTAAGGTTCCTCGCGTATCATCGAATTAAACCACATGCTCCACCGCTTGTGCGGGCCCCCGNCAATTCCTTTGAGTTTC A | *Bacteroidetes* | *Flavobacteria* | *Flavobacteriales* | *Flavobacteriaceae* | *Capnocytophaga* |
| *Dialister invisus \| HOT-118 \| clone BS095 \| AF287787 \| Named* | | TTAGAGTTTGATCCTGGCTCAGGACGAACGCTGGCGGCGTGCTTAACACATGCAAGTCGAACGAAAAGAGGGAAAGAGCTTGCTCTTTCCGGAATTGAGTGGCAAACGGGTGAGTAACACGTAAACAACCTGCCTTCAGGATGGGG ACAACAGACGGAAACGACTGCTAATACCGAATAAGTTCCAAGGGTCGCATGATCCATGGAAGAAAAGGTGGCCTCTACCTGTAAGCTATCGCCTGAAGAGGGGTTTGCGTCTGATTAGCTGGTTGGAGGGGTAACGGCCCACCAAGGCGACGATCAGTAGCCGGTCTGAGAGGATGAACGGCCACACTGGAACTGAGACACGGTCCAGACTCCTACGGGGGGCAGCAGTGGGGAATCTTCCGCAATGGGCGAAAGCCTGACGGAGCAACGCCGCGTGAGTGATGACGGCCTTCGGGTTGTAAAACTCTGTGATCCGGGACGAAAAGGCAGAGTGCGAAGAACAAACTGCATTGACGGTACCGGAAAAGCAAGCCACGGCTAACTACGTGCCAGCAGCCGCGGTAATACGTAGGTGGCAAGCGTTGTCCGGAATTATTGGGCGTAAAGCGCGCG | *Firmicutes* | *Clostridia* | *Clostridiales* | *Veillonellaceae* | *Dialister* |
| *Eikenella corrodens \| HOT-577 \| strain Moore D25 \| GQ422740 \| Named* | | TTAGAGTTTGATCCTGGCTCAGATTGAACGCTGGCGGCATGCTTTACACATGCAAGTCGAACGGCAGCGGGGTA GTGCTTGCACTACTGCCGGCGAGTGGCGAACGGGTGAGTAATATATCGGAACGTACCGAGTAATGGGGGATAACCAATCGAAAGATTGGCTAATACCGCATACGTCCTAAGGGAGAAAGCGGGGGATCGCAAGACCTCGCGTTATTCGAGCGGCCGATAACTGATTAGCTAGTTGGTGGGGTAAAGGCCTACCAAGGCGACGATCAGTAGCGGGTCTGAGAGGACGATCCGCCACACTGGGACTGAGACACGGCCCAGACTCCTACGGGAGGCAGCAGTGGGGAATTTTGGACAATGGGGGCAACCCTGATCCAGCCATGCCGCGTGTATGAAGAAGGCCTTCGGGTTGTAAAGTACTTTTGTTAGGGAAGAAAAGGGAAGTGCTAATACCACTTTTTGC TGACGGTACCTAAAGAATAAGCACCGGCTAACTACGTGCCAGCAGCCGCGGTAATACGTAGGGTGCGAGCGTTAATCGGAATTACTGGGCGTAAAGCGAGCGCAGACGGTTATTTAAGCAGGATGT | *Proteobacteria* | *Betaproteobacteria* | *Neisseriales* | *Neisseriaceae* | *Eikenella* |
| *Eubacterium [XI][G-5] saphenum \| HOT-759 \| strain ATCC 49989 \| U65987 \| Named* | | TTAGAGTTTGATCATGGCTCAGGATGAACGCTGGCGGCGTGCTTAACACATGCAAGTCGAGCGAGAAACTACATGCAGACACTTCGGTAGAAGCGAGTAGCGGAAAGCGGCGGACGGGTGAGTAACGCGTAGGCAACCTGCCCTTCACAGAGGTATAGCCTCGGGAAACCGGGATTAAAACC GCATAAAATCATAGGTTCGCATGAACCAAAGGTCAAAGATTTATCGGTGAAGGATGGGCCTGCGTCTGATTAGCTGGTTGGTAGGGTAAAAGCCTACCAAGGCGACGATCAGTAGCCGACCTGAGAGGGTGATCGGCCACATTGGAACTGAGACACGGTCCAAGCTCCTACGGGAGGCAG CAGTGGGGGATATTGCACAATGGGGGAAACCCTGATGCAGCAACGCCGCGTGAGGTATGAAGGCCTTTGGGTTGTAAGCCTCTGTCCTAGGGGAAGAAAAAAATGACGGTACCCGAGGAGGAAGCCCCGGCTAACTACGTGCCAGCAGCCGCGGTAATACGTAGGGGGCAAGCGTTATCCGGAATTATTGGGCGTAAAGAGTGCGTAGGTGGTCTACTAAGCGCGAGGTGAAAGGCAA | *Firmicutes* | *Clostridia* | *Clostridiales* | *Peptostreptococcaceae[11]* | *Eubacterium[11][G-5]* |
| *Eubacterium [XI][G-6] nodatum \| HOT-694 \| strain ATCC 33099 \| Z36274 \| Named* | | TTTACGGTTACCTTGTTACGACTTCACCCCAGTCATTGATTTCGCCTTAGGTAATCTATTGTCGACCAACTTCGGACGCCCCCAACTTCCATGGTGTGACGGGCGGTGTGTACAAGACCCGGGAACGCATTCACCGCAGCATTCTGATCTGCGATTACTAGCAACTCCGGCTTCGTGCAGGCGAGTTGCAGCCTGCAGTCCGAACTGGGACCGCCTTTTTGGTTTCGCTTAAGATCGCTCTCTCGCTTCCCTCTGTGACGGCCATTGTAGCACGTGTGTAGCCCAGAACATAAGGGGCA TGATGATTTGACGTCATCCCCGCCTTCCTCCGGGTTCTCCCCGGCAGTCCCATTAGAGTGCCCAACTTAATGATGGCAACTAACGGCAAGGGTTGCGCTCGTTGCGGGACTTAACCCAACATCTCACGACACGAGCTGACGACAACCATGCACCACCTGTCTCCACTGTTCCGAAGAAGGGCTCAGTTTTAACCGAGCTTTCAGTGGGATGTCAAGTCCTGGTAAGGTTCTTCGCGTTGCTTCGAATTAAACCACATGCTCCGCTGCTTGTGCGGGTCCCCGTCAATTCCTTTGAGTTTCA | *Firmicutes* | *Clostridia* | *Clostridiales* | *Peptostreptococcaceae[11]* | *Eubacterium[11][G-6]* |
| *Filifactor alocis \| HOT-539 \| strain ATCC 35896 \| AJ006962 \| Named* | | TTAGAGTTTGATCCTGGCTCAGGATGAACGCTGGCGGCGTGCTTAACACATGCAAGTCGAACGAACGACTATTAACAGAACCTTTCGGGGCGAAGATGATAGAAGTTAGTGGCGGACGGGTGCGTAACGCGTGGGTAATCTGCCTTTGTCAAAGGAATAACTATTCGAAAGAATAGCTAAAACCGTATAACATATTAATAGGGCATCCTAATAATATCAAAACGATAGTGGACAAAGATGA GCCCGCGTCTGATTAGCTAGTTGGTGAGATAAAAGCCCACCAAGGCAACGATCAGTAGCCGACCTGAGAGGGTGAACGGCCACATTGGAACTGAGACACGGTCCAAACTCCTACGGGAGGCAGCAGTGGGGAATATTGCACAATGGGGGGAACCCTGATGCAGCAACGCCGCGTGAGTGA AGAAGGCATTCGTGTCGTAAAACTCTGTAGTAGGGGAAGAAAGAAATGACAGTACCCTAAAAGAAAGCCCCGGCTAACTACGTGCCAGCAGCCGCGGTAATACGTAGGGGGCAAGCGTTATCCGGAATAACTGGGCGTAAAGGGTGCGCAGGTGGTTTAACAAGTTAGTGGTGAAAGGC | *Firmicutes* | *Clostridia* | *Clostridiales* | *Peptostreptococcaceae[11]* | *Filifactor* |
| *Fretibacterium sp. \| HOT-360 \| clone BH017 \| AF125199 \| Phylotype* | | TTAGAGTTTGATCATGGCTCAGGATGAACGCTGGCGGCGTGCGTAACACATGCAAGTTGAACGACGGTGCCATGAAGTGGTAACACGGAGTGGTATACGGAGTAGCGGACGGGTGAGTAAAGCATGAGAATCTGTCCTGTCGAGGGGGATAACGTTCGGAAAC GGACCCTAATACCCCATAGTCCGAGAGGTTAAAGGCAGCGATGCGCGGCAGGAGGAGCTCGTGTCCTATCAGCTGGTTGGTGAGGTAACGGCTCACCAAGGCAACGACGGGTAGCCGGCCTGAGAGGGTGACCGGCCACACTGGAACTGAGAAACGGTCCAGACTCCTACGGGAGGCAGCAGTGGGGAATATTGGGCAATGGGAGGAATCCTGACCCAGCGACGCCGCGTGAACGAAGACGGCCTTCGGGTTGTAAAGTTCTTTTATGTGGGAAGAATGAAGTGACGGTACCACATGAATAAGCCCCGGCTAACTACGTGCCAGCAGCCGCGGTAATACGTAGGGGGCGAGCGTTGTCCGGAATTACTGGGCGTAAAGGGCACGCAGGCTGTGCTTCAAGTCAGCTGTAAAAGGATGCGGCTTAACCGTGTTATGC | *Synergistetes* | *Synergistetes[C-1]* | *Synergistetes[O-1]* | *Synergistetes[F-2]* | *Fretibacterium* |
| *Fretibacterium sp. \| HOT-362 \| clone D084 \| AF125200 \| Phylotype* | | TTAGAGTTTGATCCTGGCTCAGGATGAACGCTGGCGGCGTGCGTAACACATGNAAGTTGGACGACGGTGCCATGAAGTGGTAACACGGAATGGTATACGGAGTAGCGGACGGGTGAGTAAAGCATGAGGAGCTGTCCTGTTGAGGGGGATAACGTTCGGAAACGGACCCTAATACCCCATAGGCCGAGAGGTTAAAGGCAGAGATGCGCGACAGGAGGTACTCGTGTCCTATCAGCTGGTTGGTGAGGTAACGGCTCACCAAGGCAACGACGGGTAGCCGGCCTGAGAGGGTGACCGGCCACACTGGAACTGAGACACGGTCCAGACTCCTACGGGAGGCAGCAGTGGGGAATATTGGGCAATGGGAGAAATCCTGACCCAGCGACGCCGCGTGAACGAAGACGGCCTTCGGGTTGTAAAGTTCTTTTATGTGGGAAGAAGGAAGTGACGGTACCACATGAATAAGCCCCGGCTAACTACGTGCCAGCAGCCGCGGTAATACGTAGGGGGCGAGCGTTGTCCGGAATTACTGGGCGTAAAGGGCACGCAGGCTGTGCTTCAAGTCANCTGTAAAAGGATGCGGCTTANCCGTGTTATGC | *Synergistetes* | *Synergistetes[C-1]* | *Synergistetes[O-1]* | *Synergistetes[F-2]* | *Fretibacterium* |
| *Fusobacterium naviforme \| HOT-689 \| strain DMS 20699 \| AJ006965 \| Named* | | TTAGAGTTTGATCMTGGCTCAGGATGAACGCTGNCANAATGCTTAACACATGCAAGTCAACTTGAATTTGGGTTTTTAACTTANGTTTGGGTGGAGGACGGGTGANTAACGCGTAAAGAACTTGCCTCNCNGTTANGGACGACNTTTGGAAACGAATGCTAATACCTAATATTATGATAATNNGGCATCCTATAATTATGANNGCGANWNGCGCTGTGANAGAGCTTTGCGTCCCATTAN GTNNNTGGANAGGANACGGCTCACCNNNGGGATGANGGNTAGCCGGCCTGANAGGGTGATCGGNCACGAGGGGACTGANACNCNGCCCTTACTCCTACNGGAGGCANCANTGGGGAATATTGNACAATGGACCAAGAGTCTGATCCAGCAATTCTGTGTGCNCGATGAAGTTTTTCGNANTGNAAAGTGCTTTCNNTTGGGAAGAAAGAAATGACGGNACCAACANAANAANTGACGGCTAAATACNTGCCNNCANCCGCGGNCTACNTATGTCACNANCGTTATCCGGATTTATTGGGCNTAAAGCGCTGCTAGGTGGTTATGTACNTCTGATGTGAAAATGCNAGGCTCNACTCTGTN | *Fusobacteria* | *Fusobacteria* | *Fusobacteriales* | *Fusobacteriaceae* | *Fusobacterium* |
| *Fusobacterium nucleatum ss vincentii \| HOT-200 \| clone CZ006 \| AF287810 \| Named* | | TTAGAGTTTGATCATGGCTCAGGATGAACGCTGACANAATGCTTAACACATGCAAGTCAACTTGAATTTGGGTTTTTAACTTAGGTTTGGGTGGCGGACGGGTGAGTAACGCGTAAAGAACTTGCCTCACAGCTAGGGACAACATTTGGAAACGAATGCTAATACCTGATATTATGATAATAGGGCATCCTATAATTATGAAAGCTAAAAGCGCTGTGAGAGAGCTTTGCGTCCCATTAGCTAGTTGGAGAGGTAACGGCTCACCAAGGCGATGATGGGTAGCCGGCCTGAGAGGGTGATCGGCCACAAGGGGACTGAGACACGGCCCTTACTCCTACGGGAGGCAGCAGTGGGGAATATTGGACAATGGACCAAGAGTCTGATCCAG CAATTCTGTGTGCACGATGAAGTTTTTCGGAATGTAAAGTGCTTTCAGTTGGGAAGAAAGAAATGACGGTACCAACAGAAGAAGTGACGGCTAAATACGTGCCAGCAGCCGCGGTAATACGTATGTCACGAGCGTTATCCGGATTTATTGGGCGTAAAGCGCGTCTAGGTGGTTATGTAAGTCTGATGTGAAAATGCAGGGCTCAACTCTGT | *Fusobacteria* | *Fusobacteria* | *Fusobacteriales* | *Fusobacteriaceae* | *Fusobacterium* |
| *Fusobacterium nucleatum ss vincentii \| HOT-200 \| strain ATCC 49256 \| NZ_AABF02000026 \| Named* | | TTAGAGTTTGATCCTGGCTCAGGATGAACGCTGACAGAATGCTTAACACATGCAAGTCAACTTGAATTTGGGTTTTTAACTTAGGTTTGGGTGGCGGACGGGTGAGTAACGCGTAAAGAACTTGCCTCACAGCTAGGGACAACATTTGGAAACGAATGCTAATACCTAATATTATGATAATAGGGCATCCTATAATTATGAAAGCTATAAGCGCTGTGAGAGAGCTTTGCGTCCCATTAGCTA GTTGGAGAGGTAACGGCTCACCAAGGCGATGATGGGTAGCCGGCCTGAGAGGGTGATCGGCCACAAGGGGACTGAGACACGGACCTTACTCCTACGGGAGGCAGCAGTGGGGAATATTGGACAATGGACCAAGAGTCTGATCCAGCAATTCTGTGTGCACGATGAAGTTTTTCGGAATGTAAAGTGCTTTCAGTTGGGAAGAAAGAAATGACGGTACCAACAGAAGAAGTGACGGCTAAATACGTGCCAGCAGCCGCGGTAATACGTATGTCACGAGCGTTATCCGGATTTATTGGGCGTAAAGCGCGTCTAGGTGGTTATATAAGTCTGATGTGAAAATGCAGGGCTCAACTCTGT | *Fusobacteria* | *Fusobacteria* | *Fusobacteriales* | *Fusobacteriaceae* | *Fusobacterium* |
| *Fusobacterium nucleatum ss. animalis \| HOT-420 \| strain NCTC 12276 \| X55404 \| Named* | | TTAGAGTTTGATCATGGCTCAGGATGAACGCTGACAGAATGCTTAACACATGCAAGTCTACTTGAATTTGGGTTTTTTAACTTCGATTTGGGTGGCGGACGGGTGAGTAACGCGTAAAGAACTTGCCTCACAGCTAGGGACAACATTTGGAAACGAATGCTAATACCTAATATTATGATTATAGGGC ATCCTAGAATTATGAAAGCTATATGCGCTGTGAGAGAGCTTTGCGTCCCATTAGCTAGTTGGAGAGGTAACGGCTCACCAAGGCAATGATGGGTAGCCGGCCTGAGAGGGTGAACGGCCACAAGGGGACTGAGACACGGCCCTTACTCCTACGGGAGGCAGCAGTGGGGAATATTGGACAATGGACCGAGAGTCTGATCCAGCAATTCTGTGTGCACGATGACGTTTTTCGGAATGTAAAGTGCTTTCAGTTGGGAAGAAAAAAATGACGGTACCAACAGAAGAAGTGACGGCTAAATACGTGCCAGCAGCCGCGGTAATACGTATGTCACGAGCGTTATCCGGATTTATTGGGCGTAAAGCGCGTCTAGGTGGTTATGTAAGTCTGATGTGAAAATGCAGGGCTCAACTCTG | *Fusobacteria* | *Fusobacteria* | *Fusobacteriales* | *Fusobacteriaceae* | *Fusobacterium* |
| *Fusobacterium sp. \| HOT-370 \| strain E9_12 \| AF481217 \| Unnamed* | | TTAGAGTTTGATCATGGCTCAGGATGAACGCTGNCNGAATGCTTAACACATGCNAGTCTACTTGAATTTGGGT CTTTGACTTAGATTTGGGTGGCGGACGGGTGAGTAACGCGTAAAGAACTTGCCTCACAGTTAGGGACAACATTTGGAAACGAATGCTAATACCTGATATTATGATTTTAGGGCATCCTAAGATTATGAAAGCTATATGCGCTGTGAGAGAGCTTTGCGTCCCATTAGCTAGTTGGAGAGGTAACGGCTCACCAAGGCGATGATGGGTAGCCGGCCTGAGAGGGTGAACGGCCACAAGGGGACTGAGACACGGCCCTTACTCCTACGGGAGGCAGCAGTGGGGAATATTGGACAATGGACCAAAAGTCTGATCCAGCAATTCTGTGTGCACGATGACGTTTTTCGGAATGTAAAGTGCTTTCAGTTGGGAAGAAAAAAATGACGGTACCAACAGAAGAAGTGACGGCTAAATACGTGCCAGCANCCGCGGTAATACGTATGTCACAAGCGTTATCCGGATTTATTGGGCGTAAAGCGCGTCTAGGTGGTTATGTAAGTCTGATGTGAAAATGCNGGGCTCAACTCTGT | *Fusobacteria* | *Fusobacteria* | *Fusobacteriales* | *Fusobacteriaceae* | *Fusobacterium* |
| *Haemophilus parainfluenzae \| HOT-718 \| strain CIP 102513 \| EU083530 \| Named* | | TTTACGGTTACCTTGTTACGACTTCACCCCAGTCATGAATCATACCGTGGTAAACGCCCCCCGAAGGTTAAGCTATCTACTTCTGGTACAACCCACTCCCATGGTGTGACGGGCGGTGTGTACAAGGCCCGGGAACGTATTCACCGCAACATTCTGATTTGCGATTACTAGCGATTCCGACTTCATGGAGTCGAGTTGCAGACTCCAATCCGGACTTAGACGTACTTTGTGAGATTCGCTCCAGCTCGCACTCTCGCTTCCCTCTGTATACGCCATTGTAGCACGTGTGTAGCCCTACTCGTAAGGGCCATGATGACTTGACGTCATCCCCACCTTCCTCCGGTTTATCACCGGCAGTCTCCTTTGAGTTCCCGACCTAATCGCTGGCAACAAAGGATAAGGGTTGCGCTCGTTGCGGGACTTAACCCAACATTTCACAACACGAGCTGACGACAGCCATGCAGCACCTGTCTCAGAGTTCCCGAAGGCACCAATCCATCTCTGGAATGTTCTCTGGATGTCAAGAGTAGGTAAGGTTCTTCGCGTTGCATCGAATTAAACCACATGCTCCACCGCTTGTGCGGGCCCCCGTCAATTCAT | *Proteobacteria* | *Gammaproteobacteria* | *Pasteurellales* | *Pasteurellaceae* | *Haemophilus* |
| *Lachnospiraceae [G-3] sp. \| HOT-100 \| clone EI074 \| AF385573 \| Phylotype* | | TTTAAGGNTNCCTTGNGACNACATCNCCCCCTGCATCCGCCCNGCCTTCNGGANCTCCTCCCTTGCGGNTANGTCANTGAGTTCNGTTATTGATGACTCCCNTGGGGTGGCGGGNGGNGTGNNAAACACCCGGGAACGNGGGGACCGCNNCATGCTGATGTGCNATTACTAGCGATTCCNGCTTCNAGCANGCNANGTGACCCCTGCAATCCNAGCTGAAACATGATTTTTGANATTAGCTTANNCTCGCACTCTTGCTTGTCTTTGTTTACGACNATGTCNCNCGGGTGTGANCCGAATANNANNGNGNAGANGATTAGACNTCNTCCCCATCTTCCTCCGGATTATCTCCGTCTGTCTCCTTANAGTCCCCGTCTTACTGATGGCTACNAAGGACAAGGGCTGCGCTCGTNGCGGGACTTAACCCANCATCTCACGACACGANNNNANNACNACCATGNACNCCTGNGTNNNTTGTCCCTAANGAANAANCNCNNNNCNGNTATGTNNNNGGNATGTCNAGACTTGNNAAGGTGCTANNNGTTGCTNCAAATTNAGNNACATGNNNCANCGCTTGNGCNGATCCNCGTNAAGTNNN | *Firmicutes* | *Clostridia* | *Clostridiales* | *Lachnospiraceae[14]* | *Lachnospiraceae[G-3]* |
| *Neisseria elongata \| HOT-598 \| strain ATCC 25295 \| L06171 \| Named* | | TTAGAGTTTGATCCTGGCTCAGATTGAACGCTGGCGGCATGCTTTACACATGCAAGTCGGACGGCAGCGGGGTAGTGCTTGCACTACTGCCGGCGAGTGGCGAACGGGTGAGTAATATATTGGAACGTACCGAGTAATGGGGGATAACCAATCGAAAGATTGGCTAATACCGCATACGCTCTGAGGAGGAAAGCAGGGGACCTTCGGGCCTTGCGTTATTCGAGCGGCCAATATCTGATTAGCTAGTTGGTGGGGTAAAGGCCTACCAAGGCGACGATCAGTAGCGGGTCTGAGAGGATGATCCGCCACACTGGGACTGAGACACGGCCCAGACTCCTACGGGAGGCAGCAGTGGGGAATTTTGGACAATGGGCGCAAGCCTGATCCAGCCATGCCGCGTGTCTGAAGAAGGCCTTCGGGTTGTAAAGGACTTTTGTTAGGGAAGAAAAAGGAGCGGTT AATACCCGTTTCTGCTGACGGTACCTAAAGAATAAGCACCGGCTAACTACGTGCCAGCAGCCGCGGTAATACGTAGGGTGCGAGCGTTAATCGGAATTACTGGGCGTAAAGCGAGCGCAGACGGTTACTTAAGCAGGATGT | *Proteobacteria* | *Betaproteobacteria* | *Neisseriales* | *Neisseriaceae* | *Neisseria* |
| *Peptostreptococcaceae [XI][G-7] sp. \| HOT-081 \| clone BB142 \| AF287764 \| Phylotype* | | TTAGAGTTTGATCATGGCTCAGGATGAACGCTGGCGGCATGCCTAACACATGCAAGTCGAACGAAGTCATATGTGAAGTTTTCGGATGGAATATAAGACTTAGTGGCGGACGGGTGAGTAACACGTAAGTAATCAACCTATAACACACGAATAACTAATAGAAATGTTAGCTAATACGAGATAAAATATATGATATGGGAATATTATATATCAAAGCTGAGGCGGTTATAGACGAGCTTGCGTCTGATTAGCTAGTTGGTGGGGTAAGAGCCTACCAAGGCGACGATCAGTAGCCGACCTGAGAGGGTGAACGGCCACATTGGAACTGAGAGACGGTCCAAACTCCTACGGGAGGCAGCAGTGGGGGATATTGCACAATGGGGGGAACCCTGATGCAGCAATGCCGCGTGAACGAAGAAGGCCCTAGGGTCGTAAAGTTCTGTCGTATGGGAAGAAAGAAATGACAGTACCATACAAGAAAGCCCCGGCTAACTACGTGCCAGCAGCCGCGGTAATACGTAGGGGGCAAGCGTTATCCGGAATTACTGGGCGTAAAGGGTGCGTAGGCGGCTTGTTAAGTCAAGGTTAAAAGGCAGCAGC | *Firmicutes* | *Clostridia* | *Clostridiales* | *Peptostreptococcaceae[11]* | *Peptostreptococcaceae[11][G-7]* |
| *Peptostreptococcus stomatis \| HOT-112 \| clone CK035 \| AF287763 \| Named* | | TTTACGGCTACCTTGTTACGACTTCACCCCAGTTATCGACGCCACCTTCGACGACTTCCTCCTTGCGGTTGGATAATCGGCTTCGGGTGTTTCCGACTCCCGTGGTGTGACGGGCGGTGTGTACAAGACCCGGGAACGCATTCACCGCAGCATTCTGATCTGCGATTACTAGTAACTCCAGCTTCATGTAGGCGAGTTTCAGCCTACAATCCGAACTGAGAATGGCTTTAAGGGATTGGCTCCACCTCACGGTTTGGCAACCCTCTGTACCACCCATTGTAGCACGTGTGTAGCCCTAAGCATAAGGGGCATGAT GATTTGACGTCATCCCCACCTTCCTCCAGGTTATCCCTGGCAGTCTCTCTAGAGTGCCCAACTGAATGCTGGCAACTAAAGACAAGGGTTGCGCTCGTTGCGGGACTTAACCCAACATCTCACGACACGAGCTGACGACAACCATGCACCACCTGTCACCTCAGTCCCGAAGGAAGGGTGTGATTAAACACCTGTCCGAGGGATGTCAAGCTTAGGTAAGGTTCTTCGCGTTGCTTCGAA TTAAACCACATGCTCCGCTACTTGTGCGGGTCCCCGTCAATTCCT | *Firmicutes* | *Clostridia* | *Clostridiales* | *Peptostreptococcaceae[11]* | *Peptostreptococcus* |
| *Peptostreptococcus stomatis \| HOT-112 \| strain A21H2 \| GQ422715 \| Named* | | NNGNTGTGACGGGCGGTGTGTACAAGACCCGGGAACGCATTCACCGCAGCATTCTGATCTGCNATTACTAGTAACTCCAGCTTCATGTAGGCGAGTTTCAGCCTACAATCCGAACTGAGAATGGCTTTAAGGGATTGGCTCCACCTCGCGGTTTGGCAACCCTCTGTACCACCCATTGTAGCACGTGTGTAGCCCTAAGCATAAGGGGCATGATGATTTGACGTCATCCCCACCTTCCTCCAGGTTATCCCTGGCAGTCTCTCTAGAGTGCCCAACTGAATGCTGGCAACTAAAGACAAGGGTTGCGCTCGTTGCGGGACTTAACCCAACATCTCACGACACGAGCTGACGACAACCATGCACCACCTGTCACCTCAGTCCCGAAGGAAGGGTGTGATTAAACACCTGTCCGAGGGATGTCAAGCTTAGGTAAGGTTCTTCGCGTTGCTTCGAATTAAACCACATGCTCCGCTACTTGTGCGGGTCCCCGTCAATTCCTTTGAGTTTCACACTTGCGTGCGTACTCCCCAGGCGGAGTACTTAATGCGTTAGCTGCGGCACCGAGGGGGGTAACCCCCGACACCTAGTACTCATCGTTTA | *Firmicutes* | *Clostridia* | *Clostridiales* | *Peptostreptococcaceae[11]* | *Peptostreptococcus* |
| *Porphyromonas endodontalis \| HOT-273 \| clone AJ002 \| AY005067 \| Named* | | TTTACGGTTACCTTGTTACGACTTAGCCCCAGTCACTGGTATTACCCTTAAGCGCCCCTTGCGGTTACGCTCTTCAGGTACTCCCAACTTCCATGGCTTGACGGGCGGCGTGTACAAGGCCCGGGAACGTATTCACCGCGCCATGGCTGATGCGCGATTACTAGCGAATCCAGCTTCACGGAGTCGAGTTGCAGACTCCGATCCGAACTGGGACAGGGTTTGGAGATCCGCTTCATGTCACCATGTCGCTTCCCTTTGTCCCTGCCATTGTAACACGTGTGTCGCCCCGGATGTAAGGGCCGTGCTGATTTGACGTCATCCGCCCCTTCCTCTCGTCTTACGACGGCTGTCTCGATAGAGTCCTCAGCATGACCTGTTAGTAACTATCGACGCG GGTTGCGCTCGTTATGGCACTTAAGCCGACACCTCACGGCACGAGCTGACGACAACCATGCAGCACCTACTTAGATGTCCCGAAGGAAAGCAAACTCTCATCTGCCACCATCTAAATTTCAATCCCGGGTAAGGTTCCTCGCGTATCATCGAATTAAACCACATGTTCCTCCGCTTGTGCGGGCCCCCGTCAATTCCTTTGAGTTT | *Bacteroidetes* | *Bacteroides* | *Bacteroidales* | *Porphyromonadaceae* | *Porphyromonas* |
| *Porphyromonas endodontalis \| HOT-273 \| clone P2PB_52 \| AY207054 \| Named* | | TTAGAGTTTGATCATGGCTCAGGATGAACGCTAGCGATAGGCTTAACACATGCAAGTCGAGGGGCAGCATTATTTTAGCTTGCTAAGATAGATGGCGACCGGCGCACGGGTGCGTAACGCGTATGCAACCTGCCTGTAATTAGGGAATAACCCGGTGAAAGTCGGACTAATACCCTATATTCTTCTTTCTCCGCATGGGGGAGGACGGAAAGATTTATTGATTACAGATGGGCATGCGTCCCATTAGCTGGTTGGTAAGGTAACGGCTTACCAAGGCAACGATGGGTAGGGGGACTGAGAGGTTGACCCCCCACACTGGTACTGAGACACGGACCAGACTCCTACGGGAGGCAGCAGTGAGGA ATATTGGTCAATGGGCGAGAGCCTGAACCAGCCAAGTCGCGTGAAGGACGACGGTTCTATGGATTGTAAACTTCTTTTGTAGAGGAATAATGGCAGCTACGCGTAGCTGAGATGCATGTACTCTACGAATAAGTATCGGCTAACTCCGTGCCAGCAGCCGCGGTAATACGGAGGATACGAGCGTTATCCGGAATTATTGGGTTTAAAGGGTGCGTAGGTGGCGTATTAAGTCAGTG | *Bacteroidetes* | *Bacteroides* | *Bacteroidales* | *Porphyromonadaceae* | *Porphyromonas* |
| *Porphyromonas gingivalis \| HOT-619 \| strain DSM 20709 \| X73964 \| Named* | | TTAGAGTTTGATCCTGGCTCAGGATGAACGCTAGCGATAGGCTTAACACATGCAAGTCGAGGGGCAGCATGATCTTAGCTTGCTAAGGTTGATGGCGACCGGCGCACGGGTGCGTAACGCGTATGCAACTTGCCTTACAGAGGGGGATAACCCGTTGAAAGACGGACTAAAACCGCATACACTTGTATTATTGCATGATATTACAAGGAAATATTTATAGCTGTAAGATAGGCATGCGTCCCATTAGCTAGTTGGTGAGGTAACGGCTCACCAAGGCGACGATGGGTAGGGGAACTGAGAGGTTTATCCCCCACACTGGTACTGAGACACGGACCAGACTCCTACGGGAGGCAGCAGTGAGGAATATTGGTCAATGGGCGAGAGCCTGAACCAGCCAAGTCGCGTGAAGGAAGACTGTCCTAAGGATTGTAAACTTCTTTTATACGGGAATAACGGG CGATACGAGTATTGCATTGAATGTACCGTAAGAATAAGCATCGGCTAACTCCGTGCCAGCAGCCGCGGTAATACGGAGGATGCGAGCGTTATCCGGATTTATTGGGTTTAAAGGGTGCGTAGGTTGTTCGGTAAGTCAGCGGT | *Bacteroidetes* | *Bacteroides* | *Bacteroidales* | *Porphyromonadaceae* | *Porphyromonas* |
| *Porphyromonas sp. \| HOT-395 \| clone P4GB_100 \| AY207057 \| Phylotype* | | TTAGAGTTTGATCCTGGCTCAGGATGAACGCTAGCGATAGGCTTAACACATGCAAGTCGAGGGGCAGCATTATTTTAGCTTGCTAAGATAGATGGCGACCGGCGCACGGGTGCGTAACGCGTATGCAACCTGCCTGTAATTAGGGAATAACCCGGTGAAAGTCGGACTAATACCCTATATTCTTCTTTCTCCGCATGGAGAGGGATGGAAAGATTTATTGATTACAGATGGGCATGCGTCCCATTAGCTCGTTGGTAAGGTAACGGCTTACCAAGGCAACGATGGGTAGGGGGACTGAGAGGTTGACCCCCCACACTGGTACTGAGACACGGACCAGACTCCTACGGGAGGCAGCAGTGAGGAATATTGGTCAATGGGCGGAAGCCTGAACCAGCCAAGTAGCGTGCAGGATGACGGCCCTATGGGTTGTAAACTGCTTTTATGCGGGGATAAAGGGGCTCACGTGTGGGCTTTTGCAGGTACCGCATGAATAAGGACCGGCTAATTCCGTGCCAGCAGCCGCGGTAATACGGAAGGTCCCGGCGTTATCCGGATTTATTGGGTTTAAAGGGAGCGTAGGCCGTGGATTAAGCGTGTTGT | *Bacteroidetes* | *Bacteroides* | *Bacteroidales* | *Porphyromonadaceae* | *Porphyromonas* |
| *Prevotella loescheii \| HOT-658 \| strain ATCC 15930 \| L16481 \| Named* | | TTAGAGTTTGATCATGGCTCAGGATGGACGCTAGCTACAGGCTTAACACATGCAAGTCGCGGGGCAGCATGGGGGTTGCTTGCAACTCCCGATGGCGACCGGCGCACGGGTGAGTAACGCGTATCCAACCTGCCCTTCACCACGGGATAACCCGGCGAAAGTCGGACTAATACCGTATGTTGTCCATTGACGGCATCCGATTTGGACGAAAGGCTTTGCGGTGAAGGATGGGGATGCGTCCGATTAGCCAGACGGCGGGGTAACGGCCCACCGTGGCTACGATCGGTAGGGGTTCTGAGAGGAAGGTCCCCCACACTGGAACTGAGACACGGTCCAGACTCCTACGGGAGGCAGCAGTGAGGAATATTGGTCAATGGGCGTAAGCCTGAACCAGCCAAGTAGCGTGCAGGATGACGGCCCTATGGGTTGTAAACTGCTTTTATGCGGGGATAAAGTGGCCCACGTGTGGGTTTTTGCAGGTACCGCATGAATAAGGACCGGCTAATTCCGTGCCAGCAGCCGCGGTAATACGGAAGGTCCGGGCGTTATCCGGATTTATTGGGTTTAAAGGGAGCGTAGGCCGCGCCTTAAGCGTGTTGT | *Bacteroidetes* | *Bacteroides* | *Bacteroidales* | *Prevotellaceae* | *Prevotella* |
| *Prevotella pallens \| HOT-714 \| strain 9423 \| Y13106 \| Named* | | TTAGAGTTTGATCCTGGCTCAGGATGAACGCTAGCTATAGGCTTAACACATGCAAGTCGAGGGGAAACGGCATTATGTGCTTGCACATTTTGGACGTCGACCGGCGCACGGGTGAGTATCGCGTATCCAACCTGCCCTTTACTTGGGGATACCCCGTTGAAAGACGGCCTAATACCCGATGTAATTCATTGATGGCCTCAGATATGAATAAAAGATTTATCGGTAAAGGATGGGGATGCGTCTGATTAGCTTGTTGGTGAGGTAATGGCTCACCAAGGCGACGATCAGTAGGGGTTCTGAGAGGAAGGTCCCCCACATTGGAACTGAGACACGGTCCAAACTCCTACGGGAGGCAGCAGTGAGGAATATTGGTCAATGGACGTAAGTCTGAACCAGCCAAGTAGCGTGCAGGAAGACGGCCCTATGGGTTGTAAACTGCTTTTATACGAGAATAATTT GATGCACGTGTGCGTTATTGCATGTATCGTATGAATAAGGACCGGCTAATTCCGTGCCAGCAGCCGCGGTAATACGGAAGGTCCAGGCGTTATCCGGATTTATTGGGTTTAAAGGGAGTGTAGGCGGTTTGTTAAGCGTGTT | *Bacteroidetes* | *Bacteroides* | *Bacteroidales* | *Prevotellaceae* | *Prevotella* |
| *Prevotella sp. \| HOT-310 \| clone DR022 \| GQ422737 \| Phylotype* | | TTTACGGTTACCTTGTTACGACTTAGCCCCAATTACCAGTTTTGCCCTAGGTCGCT CCTTACGGTTACGAACTTCAGGCACCCCCGGCTTTCATGGCTTGACGGGCGGTGTGTACAAGGCCCGGGAACGTATTCACCGCGCCATGGCTGATGCGCGATTACTAGCGAATCCAGCTTCGTGAGGTCGGGTTGCAGACCTCAGTCCGAACTGGGACCGGCTTTTAAGATTTGATGCAATTTGCATTACACCATCCCTCTGTACCGGCCATTGTAACACGTGTGTAGCCCCGGACGTAA GGGCCGTGCTGATTTGACGTCATCCCCACCTTCCTCACACCTTGCGGTGGCAGTGTTCCCAGAGTGCCCAGCATTACCTGATGGCAACTAAGAAAAGGGGTTGCGCTCGTTATGGCACTTAAGCCGACACCTCACGGCACGAGCTGACGACAACCATGCAGCACCTTCACAGAGACCCCGAAGGGCTTATTTATCTCTAAATAATTCCTCTGCAATTCAAGCCCGGGTAAGGTTCCTCGC GTATCATCGAATTAAACCACATGTTCCTCCGCTTGTGCGGGCCCCCGTCAATTCCTTTGAGTTT | *Bacteroidetes* | *Bacteroides* | *Bacteroidales* | *Prevotellaceae* | *Prevotella* |
| *Prevotella sp. \| HOT-317 \| clone C3MKM081 \| AY278624 \| Unnamed* | | TTAGAGTTTGATCATGGCTCAGGATGAACGCTAGCTACAGGCTTAACACATGCAAGTCGCGGGGCAGCATGGGGGTTGCTTGCAATCCCTGATGGCGACCGGCGCACGGGTGAGTAACGCGTATCCAACCTGCCCTTTACCACGGGATAACCCGGCGAAAGTCGGACTAATACCGTATGTTGTCCATTGACGGCATCCGATTTGGACGAAAGGCTTAGCGGTGAGGGATGGGGATGCGTCCGATTAGCTCGACGGCGGGGTAACGGCCCACCGTGGCTACGATCGGTAGGGGTTCTGAGAGGAAGGTCCCCCACACTGGAACTGAGACACGGTCCAGACTCCTACGGGAGGCAGCAGTGAGGAATATTGGTCAATGGGCGGAAGCCTGAACCAGCCAAGTAGCGTGCAGGATGACGGCCCTATGGGTTGTAAACTGCTTTTATGCGGGGATAAAGGAGCCCACGTGTGGGTTTTTGCAGGTACCGCATGAATAAGGACCGGCTAATTCCGTGCCANCAGCCGCGGTAATACGGAAGGTCCGGGCGTTATCCGGATTTATTGGGTTTAAAGGGAGCGTANGCCGCCCCTTAAGCGTGTTGT | *Bacteroidetes* | *Bacteroides* | *Bacteroidales* | *Prevotellaceae* | *Prevotella* |
| *Prevotella sp. \| HOT-317 \| strain B31FD \| AY005061 \| Unnamed* | | TTAGAGTTTGATCCTGGCTCAGGATGAACGCTAGCTACAGGCTTAACACATGCAAGTCGCGGGGCAGCATGGGGGTTGCTTGCAATCCCTGATGGCGACCGGCGCACGGGTGAGTAACGCGTATCCAACCTGCCCTTCACCACGGGATAACCCGGCGAAAGTCGGACTAATACCGTATGTTGTCCATTGACGGCATCCGATTTGGACGAAAGGCTTTGCGGTGAGGGATGGGGATGCG TCCGATTAGCTCGACGGCGGGGTAACGGCCCACCGTGGCTACGATCGGTAGGGGTTCTGAGAGGAAGGTCCCCCACACTGGAACTGAGACACGGTCCAGACTCCTACGGGAGGCAGCAGTGAGGAATATTGGTCAATGGGCGGAAGCCTGAACCAGCCAAGTAGCGTGCAGGATGACGGCCCTATGGGTTGTAAACTGCTTTTATGCGGGGATAAAGGAGCCCACGTGTGGGTTTTTGCAGGTACCGCATGAATAAGGACCGGCTAATTCCGTGCCAGCAGCCGCGGTAATACGGAAGGTCCGGGCGTTATCCGGATTTATTGGGTTTAAAGGGAGCGTAGGCCGCCCCTTAAGCGTGTT GT | *Bacteroidetes* | *Bacteroides* | *Bacteroidales* | *Prevotellaceae* | *Prevotella* |
| *Prevotella sp. \| HOT-443 \| clone P4PB_24 \| AY331415 \| Phylotype* | | TAGGCCGATCCTTCCGGTCACGGACTTCAGGCACCCCCGGCTTTCATGGNTTGACGGGCGGTGTGTACAAGGC CCGGGAACGTATTCACCGCGCCATGGCTGATGCGCGATTACTAGCGAATCCAGCTTCGTGGGGTCGGGTTGCAGACCCCAGTCCGAACTGGGACCGGATTTAAAGATTAGATCGCATTTGCATACGACCGACCTTCTGTACCGGCCATTGNAACACGTGTGTAGCCCCGGACGTAAGGGCCGTGCTGATTTGACGTCATCCCCACCTTCCTCACACCTTACGGCGGCAGTATCTACAGAGTGCCCGGCATTACCCGATGGCAACTGAAGAAAGGGGTTGCGCTCGTTATGGCACTTAAGCCGACACCTCACGGCACGAGCTGACGACAACCATGCAGCACCTCCGCGGCAGCCCCGAAGGGCCTCATCCTCTCGGANTCGTTCCGCCGCANTTCAAGCCCGGGTAANGNTCCTCGCGTATCATCGAATTAAACCACATGTTCCTCCGCTTGTGCGGGCCCCGTNANTCCTTTGAGTTTCA CCGTTGCCGGCGTACTCCCCAGGNGGGATGCTTAATGCTTTCGCTGG | *Bacteroidetes* | *Bacteroides* | *Bacteroidales* | *Prevotellaceae* | *Prevotella* |
| *Prevotella sp. \| HOT-472 \| clone GU027 \| AY349398 \| Unnamed* | | TTAGAGTTTGATCATGGCTCAGGATGGACGCTAGCTACAGGCTTAACACATGCAAGTCGCGGGGCAGCATGGGGGTTGCTTGCAACTCCCGATGGCGACCGGCGCACGGGTGAGTAACGCGTATCCAACCTGCCCTTCACCACGGGATAACCCGGCGAAAGTCGGACTAATACCGTATGTTGTCCATTGACGGCATCCGATTTGGACGAAAGGCTTTGCGGTGAAGGATGGGGATGCG TCCGATTAGCCAGACGGCGGGGTAACGGCCCACCGTGGCTACGATCGGTAGGGGTTCTGAGAGGAAGGTCCCCCACACTGGAACTGAGACACGGTCCAGACTCCTACGGGAGGCAGCAGTGAGGAATATTGGTCAATGGGCGTAAGCCTGAACCAGCCAA GTAGCGTGCAGGATGACGGCCCTATGGGTTGTAAACTGCTTTTATGCGGGGATAAAGTGGCCCACGTGTGGGTTTTTGCAGGTACCGCATGAATAAGGACCGGCTAATTCCGTGCCAGCAGCCGCGGTAATACGGAAGGTCCGGGCGTTATCCGGATTTATTGGGTTTAAAGGGAGCGTAGGCCGCGCCTTAAGCGTGTTGT | *Bacteroidetes* | *Bacteroides* | *Bacteroidales* | *Prevotellaceae* | *Prevotella* |
| *Prevotella sp. \| HOT-515 \| clone MB3_P13 \| DQ003622 \| Phylotype* | | TTAGAGTTTGATCATGGCTCAGGATGAACGCTGGCTACAGGCTTAACACATGCAAGTCGAGGGGCATCACGGGGGTTGCTTGCAACCCCTGGTGGCGACCGGCGAATGGGTGAGTAACGCGTATCCAACCTGCCCCCGGCCGGGGNATAACCCGTCGAAAGGNGGCCTAATCCCCCATGCCGTCCTCCGCGGCCATCCAAGGAGGACGAAAGGTATTTTCCGGCCGGGCGATGGGGATGCGTCCGATTAGCTCGCTGGCGGGGTAACGGCCCACCAGGGCTTCGATCGGTAGGGGTTCTGAGAGGAAGGTCCCCCACACTGGTACTGAGACACGGACCAGACTCCTACGGGAGGCAGCAGTGAGGAATATTGGTCAATGGACGTAAGTCTGAACCAGCCAAGTAGCGTGCAGGATGACG GCCCTATGGGTTGTAAACTGCTTTTATGCGGGGATAAAGGAGCCCACGTGTGGGTTTTTGCAGGTACCGCATGAATAAGGACCGGCTAATTCCGTGCCAGCAGCCGCGGTAATACGGAAGGTCCGGGCGTTATCCGGATTTATTGGGTTTAAAGGGAGCGTANNCCGCCCCTTAAGCGTGT | *Bacteroidetes* | *Bacteroides* | *Bacteroidales* | *Prevotellaceae* | *Prevotella* |
| *Prevotella sp. \| HOT-526 \| strain P4P_53 \| AY944134 \| Unnamed* | | TTAGAGTTTGATCCTGGCTCAGGATGAACGCTAGCTACAGGCTTAACACATGCAAGTCGAGGGGCATCATGCGGATTGCTTGCGATCCGTGATGGCGACCGGCGCACGGGTGAGTAACGCGTATCCAACCTTCCCGCTGCTGGGGGGATAACCNGGTGAAAGCCGGACTAATACCCCATAATCCCCGTTGACGGCATCCGATTCGGGGCAAAGTTTTTTTTCGGCAGCGGATGGGGATGC GTCCGATTAGTTAGTTGGTGAGGTAACGGCTCANCAAGGCTTTGATCGGNAGGGGTTCTGANANGA | *Bacteroidetes* | *Bacteroides* | *Bacteroidales* | *Prevotellaceae* | *Prevotella* |
| *Selenomonas artemidis \| HOT-124 \| clone AA024 \| AF287797 \| Named* | | TTTACGGTTACCTTGTTACGACTTCACCCCAGTCACCTTCCCCACCTTAGACGGCTGTGCCGGCTTCGGGTGTGAACGACTTCCGTGGTGTGACGGGCGGTGTGTACAAGACCCGGGAACGTATTCACCGCAGTATGCTGACCTGCGATTACTAGCGATTCCGACTTCATGCAGGCGAGTTGCAGCCTGCAATCCGAACTGAGAAACGGTTTTTGAGGTTCGCTTAAGATCGCTCTCTTGCTGCTCTCTGTCCGTCCCATTGTAGTACGTGTGTAGCCCAGACCATAAGGGGCATGATGACTTGACGTCATCCCCGCCTTCCTCCGCGTTCTCCGCGGCAGTCTCCTTTGAGTGCCCACCTTAAATGCTGGCAACAAAGGACAGGGGTTGCGCTCGTTGCGGGACTTAACCCAACATCTCACGACACGAGCTGACGACAACCGTGCACCACCTGTTTTCTGGCTTCCGAAGAAGAGGAACTATCTCTAGTTCTGTCCATCAATGTCAAGACCTGGTAAGGTTCTTCGCGTTGCGTCGAATTAAACCACATACTCCACCGCTTGTGCGGGCCCCCGTCAATTCCTTTGAGTTTCAACCTT | *Firmicutes* | *Clostridia* | *Clostridiales* | *Veillonellaceae* | *Selenomonas* |
| *Selenomonas artemidis \| HOT-124 \| strain ATCC 43528 \| GQ422716 \| Named* | | TTAGAGTTTGATCCTGGCTCAGGACGAACGCTGGCGGCGTGCTTAACACATGCAAGTCGAACGGAGCGAATGAAAGCTTGCTTTTATGAGCTTAGTGGCAAACGGGTGAGTAACACGTAGACAACCTGCCGATAGGATGGGGACAACATTCCGAAAGGAATGCTAATACCGAATGGCGTGTA GGGGAGGCACCTCCCGTACATGAAAGATGGCCTCTGAATATGCTATCACCTATCGATGGGTCTGCGTCTGATTAGCTGGTAGGTAAGGTAACGGCTTACCTAGGCGACGATCAGTAGCCGGTCTGAGAGGATGAACGGCCACATTGGGACTGAGACACGGCCCAGACTCCTACGGGAGGC AGCAGTGGGGAATCTTCCGCAATGGGCGCAAGCCTGACGGAGCAACGCCGCGTGAGTGAAGAAGGTCTTCGGATCGTAAAGCTCTGTTGAAGGGGACGAACGATCGAGGGGCGAACAGGCTCTCGGTATGACGGTACCTTTTGAGGAAGCCACGGCTAACTACGTGCCAGCAGCCGCGGT AATACGTAGGTGGCGAGCGTTGTCCGGAATCATTGGGCGTAAAGGGAGCGCAGGCGGC | *Firmicutes* | *Clostridia* | *Clostridiales* | *Veillonellaceae* | *Selenomonas* |
| *Selenomonas infelix \| HOT-639 \| strain ATCC 43532 \| AF287802 \| Named* | | TTAGAGTTTGATCATGGCTCAGGACGAACGCTGGCGGCGTGCTTAACACATGCAAGTCNAACGGAGCGAATGAAAGCTTGCTTTTATGAGCTTAGTGGCAAACGGGTGAGTGACACGTAGACAACCTGCCGACAGGATGGGGACAACATTCCGAAAGGAATGCT AATACCGAATGAAGCGNAGGAGAGGCATCTCTCCTCCGTGAAAGATGGCCTCTATTTATAAGCTATCACCTGTCGATGGGTCTGCGTCTGATTAGCTAGTTGGTGAGGTAACGGCTCACCAAGGCGACGATCAGTAGCCGGTCTGAGAGGATGAACGGCCACATTGGGACTGAGACACGGCCCAGACTCCTACGGGAGGCAGCAGTGGGGAATCTTCCGCAATGGGCGCAAGCCTGACGGAGCAACGCCGCGTGAGTGAAGAAGGTCTTCGGATCGTAAAGCTCTGTTGATGGGGACGAACGTGCGAAGGGTGAATAATCCTTTGCAATGACGGTACCTATCGAGGAAGCCACGGCTAACTACGTGCCAGCAGCCGCGGTAATACGTAGGTGGCGAGCGTTGTCCGGAATCATTGGGCGTAAAGGGAGCGCAGGCG | *Firmicutes* | *Clostridia* | *Clostridiales* | *Veillonellaceae* | *Selenomonas* |
| *Selenomonas sp. \| HOT-143 \| clone EW051a \| AF385497 \| Phylotype* | | TTTACGGCTACNNNNNNNNNNNNCACCCCAGTCATCGCCCCCGCCTTAGACGNNGCCTCCTTGCGGTTGGCCCACCGGCTTTGGGCGTGAATGACTTCCGTGGTGTGACGGGCGGTGTGTACAAGGCCCGGGAACGTATTCACCGCAGTATGCTGACCTGCGATTACTAGCGATTCCGACTTCATGCAGGCGGGTTGCAGCCTGCAATCCGAACTGGGGGATGGTTTGTGGGGTCCGCT CCGGCTCGCGCCTTCGCTTCCCTCTGTCCATCCCATTGTAGTACGTGTGTAGCCCAGGACATAAGGGGCATGATGACTTGACGTCATCCCCGCCTTCCTCCGCGTTCTCCGCGGCAGTCTCCTTTGAGTTCCCGCCATTACGCGCTGGCAACAAAGGACAGGGGTTGCGCTCGTTGCGGGACTTAACCCAACATCTCACGACACGAGCTGACGACAGCCATGCACCACCTGTTTTCGTGTTCCCGAAGGAAGGGAGCTATCTCTAGCTCTTTCACTCAATGTCAAGCCCTGGTAAGGTTC TTCGCGTTGCGTCGAATTAAACCACATACTCCACCGCTTGTGCGGGCCCCCGTCAATTCCT | *Firmicutes* | *Clostridia* | *Clostridiales* | *Veillonellaceae* | *Selenomonas* |
| *Selenomonas sp. \| HOT-892 \| strain F0426 \| tbd \| Unnamed* | | TTAGAGTTTGATCCTGGCTCAGGACGAACGCTGGCGGCGTGCTTAACACATGCAAGTCGAACGGAGCGAATGAAAGCTTGCTTTTATGAGCTTAGTGGCAAACGGGTGAGTAACACGTAGACAACCTGCCGACAGGATGGGGACAACATTCCGAAAGGAATGCTAATACCGAATGAAGTCGAGGGAAGGCATCTTCCTTCGATGAAAGATGGCCTCTGTTTATAAGCTATCACCTGTCGATGGGTCTGCGTCTGATTAGCTGGTTGGTGAGGTAACGGCTCACCAAGGCGACGATCAGTAGCCGGTCTGAGAGGATGAACGGCCACATTGGGACTGAGACACGGCCCAAACTCCTACGGGAGGCAGCAGTGGGGAATATTGGACAATGGGGGCAACCCTGA TCCAGCGACGCCGCGTGAGTGAAGAAGTATTTCGGTATGTAAAGCTCTATCGATAACGGAAGAAGATGACAAGCCGTTAAGGAAGAAGCCCCGGCTAACTACGTGCCAGCAGCCGCGGTAATACGTAGGGGGCAAGCGTTATCCGGATTTACTGGGTGTAAAGGGAGCGTAGACGGCGAATAAAGTCTGAAGTGAAATC | *Firmicutes* | *Clostridia* | *Clostridiales* | *Veillonellaceae* | *Selenomonas* |
| *Selenomonas sputigena \| HOT-151 \| strain ATCC 35185 \| AF287793 \| Named* | | TTTACGGCTACCTTGTTACGACTTCACCCCAGTCATCGCCCCCGCCTTAGACGGCTGCCTCCTTGCGGTTGGCCCACCGGCTTTGGGCGTGAATGACTTCCGTGGTGTGACGGGCGGTGTGTACAAGGCCCGGGAACGTATTCACCGCAGTATGCTGACCTGCGATTACTAGCGATTCCGACTTCATGCAGGCGGGTTGCAGCCTGCAATCCGAACTGGGGGATGGTTTGTGGGG | *Firmicutes* | *Clostridia* | *Clostridiales* | *Veillonellaceae* | *Selenomonas* |
| *Streptococcus anginosus \| HOT-543 \| strain ATCC 33397 \| AF104678 \| Named* | | TTTACGGTTACCTTGTTACGACTTCACCCCAATCATCTATCCCACCTTAGGCNNCTGGCTCCTTACGGTTACCTCACCGACTTCGGGTGTTACAAACTCTCGTGGTGTGACGGGCGGTGTGTACAAGGCCCGGGAACGTATTCACCGCGGCGTGCTGATCCGCGAT TACTAGCGATTCCGACTTCATGTAGGCGAGTTGCAGCCTACAATCCGAACTGAGACTGGCTTTCAGAGATTAGCTTGNCGTCACCGGCTTGCGACTCGTTGTACCAGCCATTGTAGCACGTGTGTAGCCCAGGTCATAAGGGGCATGATGATTTGACGTCATCCCCACCTTCCTCCGGTTTATTACCGGCAGTCTCGCTAGGGTGCCCAACTCAATGATGGCAACTAACAATAAGGGTTGCGCTCGTTGCGGGACTTAACCCAACATCTCACGACACGAGCTGACGACAACCATGCACCACCTGTCACCGATGTTCCGAAGAAACTTCCTATCTCTAGAAATAGCATCGGGATGTCAAGANCTGGTAAGGTTCTTCGCGTTGCTTCNAATTAAACCACATGCTCCACCGCTTGTGCGGGCCCCCGTCAATTCCT | *Firmicutes* | *Bacilli* | *Lactobacillales* | *Streptococcaceae* | *Streptococcus* |
| *Streptococcus mitis bv 2 \| HOT-398 \| strain SK34 \| AY005045 \| Named* | | TTAGAGTTTGATCNTGGCTCAGGACGAACGCTGGCGGCGTGCCTAATACATGCAAGTAGAACGCTGAAGGAGGAGCTTGCTTCTCTGGATGAGTTGCGAACGGGTGAGTAACGCGTAGGTAACCTGCCTGGTAGCGGGGGATAACTATTGGAAACGATAGCTAATACCGCATAATAGCAGTTATTGCATGATAACTGTTTGAAAGGTGCAATTGCACCACTACCAGATGGACCTGCGTT GTATTAGCTAGTTGGTGGGGTAACGGCTCACCAAGGCGACGATACATAGCCGACCTGAGAGGGTGATCGGCCACACTGGGACTGAGACACGGCCCAGACTCCTACGGGAGGCAGCAGTAGGGAATCTTCGGCAATGGACGGAAGTCTGACCGAGCAACGCCGCGTGAGTGAAGAAGGTTT TCGGATCGTAAAGCTCTGTTGTAAGAGAAGAACGAGTGTGAGAGTGGAAAGTTCACACTGTGACGGTATCTTGCCGGAAAGGGACGGCTAACTACGTGCCAGCAGCCGCGGTAATACGTAGGTCCCGAGCGTTGTCCGGATTTATTGGGCGTAAAGCGAGCGCAGGCGGTTAGATAAGTCT | *Firmicutes* | *Bacilli* | *Lactobacillales* | *Streptococcaceae* | *Streptococcus* |
| *Streptococcus sanguinis \| HOT-758 \| strain ATCC 10556 \| AF003928 \| Named* | | TTAGAGTTTGATNCTGGCTCAGGACGAACGCTGGCGGCGTGCCTAATACATGCAAGTAGAACGCTGAAGAGAGGAGCTTGCTCTTCTTGGATGAGTTGCGAACGGGTGAGTAACGCGTAGGTAACCTGCCTGGTAGCGGGGGATAACTATTGGAAACGATAGCTAATACCGCATGATATTAATTATCGCATGATAATTGATTGAAAGATGCAATTGCATCACTACCAGATGGACCTGCGTTGTATTAGCTAGTTGGTGAGGTAACGGCTCACCAAGGCGACGATACATAGCCGACCTGAGAGGGTGATCGGCCACACTGGGACTGAGACACGGCCCAGACTCCTACGGGAGGCAGCAGTAGGGAATCTTCGGCAATGGGGGGAACCCTGACCGAGCAACGCCGCGTGAGTGAAGAAGGTTTTCGGATCGTAAAGCTCTGTTGTAAGAGAAGAACGGGTGTGAGAGTGGAAAGTTCACACTGTGACGGTATCTTACCAGAAAGGGACGGCTAACTACGTGCCAGCAGCCGCGGTAATACGTAGGTCCCGAGCGTTGTCCGGATTTATTGGGCGTAAAGCGAGCGCAGGCGGTTAGATAAGT | *Firmicutes* | *Bacilli* | *Lactobacillales* | *Streptococcaceae* | *Streptococcus* |
| *Streptococcus sp. \| HOT-071 \| strain Hans H6 \| AY005041 \| Unnamed* | | TTAGAGTTTGATCATGGCTCAGGACGAACGCTGGCGGCGTGCCTAATACATGCAAGTAGAACGCTGAAGAGAGGAGCTTGCTCTTCTTGGATGAGTTGCGAACGGGTGAGTAACGCGTAGGTAACCTGCCTGGTAGCGGGGGGATAACTATTGGAAACGATAGCTAATACCGCATAAAATGGATTATCGCATGATAATCAATTGAAAGGTGCAAATGCATCACTACCAGATGGACCTGC GTTGTATTAGCTAGTTGGTGGGGTAACGGCTCACCAAGGCGACGATACATAGCCGACCTGAGAGGGTGATCGGCCGCACTGGGACTGAGACACGGCCCAGACTCCTACGGGAGGCAGCAGTAGGGAATCTTCGGCAATGGACGGAAGTCTGACCGAGCAACGCCGCGTGAGTGAAGAAGG TTTTCGGATCGTAAAGCTCTGTTGTAAGANAAGAACGAGTGTGAGAGTGGGAAGTTCACACTGTGACGGTATCTTACCANAAAGGGACGGCTAACTACGTGCCANCAGCCGCGGTAATACGTANGTCCCGAGCGTTGTCCGGATTTATTGGGCGTAAAGCGAGCGCANGCGGTTANATAAG | *Firmicutes* | *Bacilli* | *Lactobacillales* | *Streptococcaceae* | *Streptococcus* |
| *Tannerella forsythia \| HOT-613 \| strain FDC 338 \| L16495 \| Named* | | TTTACGGTTACCTTGTTACGACTTAGCCCCAGNCACCTGTATTACCCTAGGCCGACCCTCGCGGTTACGGACTTTAGGTACTCCAGACTCCCATGGCTTGACAGGCGGTGTGTACAAGGCCCGGGAACGTATTCACCGCGCCATGGCTGATGCGCGATTACTAGCGAATCCAGCTTCACGGAGTCGAGTTGCAGACTCCGATCCGAACTGAGACAGGGTTTAGAGATTTGCATCCGATCGCTCGGTAGCTGCCCTTTGTCCCTGCCATTGTAACACGTGTGTCGCCCCGGATGTAAGGGCCGTGCTGATTTGACGTC ATCCCAACCTTCCTCACAGCTTACGCCGGCAGTCCCGCCAGAGTCCTCAGCTTAACCTGTTAGCAACTGACAGTCAGGGTTGCGCTCGTTATGGCACTTAAGCCGACACCTCACGGCACGAGCTGACGACAACCATGCAGCACCTACTTAGATGTCCCGAAGGAAAGCAAACTCTCATCTGCCACCATCTAAATTTCAATCCCGGGTAAGGTTCCTCGCGTATCATCGAATTAAACCACATGTTCCTCCGCTTGTGCGGGCCCCCGTCAATTCCTTTGAGTT | *Bacteroidetes* | *Bacteroides* | *Bacteroidales* | *Porphyromonadaceae* | *Tannerella* |
| *Tannerella sp. \| HOT-286 \| clone BU063 \| AY008308 \| Phylotype* | | TTTACGGTTACCTTGTTACGACTTAGCCCCAGTCACCAGTTTTACCCTAGGCCGATCCTCAACGGTTACGGACTTTAGGCACCCCCGGCTCCCATGGCTTGACGGGCGGTGTGTACAAGGCCCGGGAACGTATTCACCGCGCCATGGCTGATGCGCGATTACTAGCGAATCCAGCTTCACGGAGTCGAGTTGCAGACTCCGATCCGAACTGAGACAGGGTTTAGAGATTTGCATCCGATCGCTCGGTAGCTGCCCTTTGTCCCTGCCATTGTAACACGTGTGTCGCCCCGGATGTAAGGGCCGTGCTGATTTGACGTCATCCCAACCTTCCTCACAGCTTACGCCGGCAGTCCCGCCAGAGTCCTCAGCTTAAC CTGTTAGCAACTGACAGTCAGGGTTGCGCTCGTTATGGCACTTAAGCCGACACCTCACGGCACGAGCTGACGACAGCCATGCAGCACCTGTGTTACGGCTCCCGAAGGCACTCCTCCGTCTCTGGAGGATTCCGTACATGTCAAGACCAGGTAAGGTTCTTCGCGTTGCATCGAATTAATCCACATCATCCACCGCTTGTGCGGGTCCCCGTCAATTCCTTTGAG | *Bacteroidetes* | *Bacteroides* | *Bacteroidales* | *Porphyromonadaceae* | *Tannerella* |
| *Terrahaemophilus aromaticivorans \| HOT-826 \| strain 127W \| AB098612 \| Named* | | TTAGAGTTTGATCATGGCTCAGATTGAACGCTGGCGGCAGGCTTAACACATGCAAGTCGAACGGTAACATAAAGAAGCTTGCTTCTTTGATGACGAGTGGCGGACGGGTGAGTAATGCTTGGGAATCTAGCTTATGGAGGGGGATAACTACGGGAAACTGTAGCTAATACCGCGTAGAATCGAGAGATGAAAGTGTGGGACCTTCGGGCCACATGCCATAGGATGAGCCCAAGTGGGATTAGGTAGTTGGTGAGGTAAAGGCTCACCAAGCCGACGATCTCTAGCTGGTCTGAGAGGATGACCAGCCACACTGGGACTGAGACACGGCCCAGACTCCTACGGGAGGCAGCAGTGGGGAATATTGCGCAATGGGGGCAACCCTGACGCGGCCATGCCGCGTGAATGAAGAAGGCCTTCGGGTTGTAAAGTTCTTTCGGTAGCGAGGAAGGCATTTAGTTTAATAGACTAGGTGATTGACGTTAACTACAGAAGAAGCACCGGCTAACTCCGTGCCAGCAGCCGCGGTAATACGGAGGGTGCGAGCGTTAATCGGAATAACTGGGCGTAAAGGGCACGCAGGCGGTGACTTAAGTGAGGTGT | *Proteobacteria* | *Gammaproteobacteria* | *Pasteurellales* | *Pasteurellaceae* | *Terrahaemophilus* |
| *Treponema lecithinolyticum \| HOT-653 \| clone GF6 \| AF023040 \| Named* | | TTTACGGCTACCTTGTTACGACTTCACCCTCCTTANNAANCATACCTTCGGCAGCNTCCTCCTTGCGGTTAGACTACCGACTTCGGGTACCCTCNACTCNGATGGTGTGACGGGCGGTGTGTACAAGGCCCGGGAACGTATTCACCGCATCNTGCTGATATGCGATTACTAGCGATTCCAACTTCATGGAGNCGAGTTKCAGACTCCAATCCGAACTACGATAGCTTTTCTGCGTTTTGCTCC ACCTCGCGGCTTCGCTTCACTCTGTCGCTACCATTGTAGCACGTGTGTAGCCCTGGACATAAGGGCCATGATGACTTGACGTCATCCCCACCTTCCTCCGGTTTGTCACCGGCAGTTCCGCCAGAGTCCTCAAGCATTACCTGTTAGTAACTGGCAGTAGGGGTTGCGCTCGTTGCGGGACTTAACCCAACACCTCACGGCACGAGCTGACGACAGCCATGCAGCACCTGTTGTACTTCGTATTGCTACGCATCCGTATCTCTACGAACTTAAGTACTATGTCAAACCCAGGTAANGNTCCTCGCGTACCATCGAATTAAACCACATGCTCCACCGCTTGTGCNGGCCCCCGTCNAT | *Spirochaetes* | *Spirochaetes* | *Spirochaetales* | *Spirochaetaceae* | *Treponema* |
| *Treponema medium \| HOT-667 \| clone _C007 \| AF023051 \| Named* | | TTTACGGCTACCTTGTTACGACTTCACCCTCCTTACCAAGCGTACCTTCGGCACCGTCCTCCTTTGCAGGTTAGACAAGCGACTTCGGGTACCCCCAACTCGGATGGTGTGACGGGCGGTGTGTACAAGGCCCGGGAACGTATTCACCGCGCCGTGCTGATGCGCGATTACTAGCGATTCCAACTTCATGAAGTCGAGTTTCANACTTCAATCCGAACTACGATTGCTTTTTTGCGGTTTGCTCCACCTTACGGTCTTGCTTCGCTTTGTAGCAACCATTGTAGCACGTGTGTAGCCCTG GACATAAGGGGCATGATGACTTGACGTCATCCCCGCCTTCCTCCGCGTTCTCCGCGGCAGTCTCCTTTGAGTGCCCAGCTTTACCTGATGGCAACAAAGAACAGGGGTTGCGCTCGTTGCGGGACTTAACCCAACATCTCACGACACGAGCTGACGACAACCATGCACCACCTGTCTCCTCTGTCCGAAGAAATACCCGATTAAGGGTATGTCAGAGGGATGTCAAGTCTTGGTAAGGTT CTTCGCGTTGCTTCGAATTAAACCACATGCTCCGCTGCTTGTGCGGGTCCCCGTCAATTC | *Spirochaetes* | *Spirochaetes* | *Spirochaetales* | *Spirochaetaceae* | *Treponema* |
| *Treponema sp. \| HOT-270 \| clone DD012 \| GQ422733 \| Phylotype* | | TTNNNGTTTGCTTTGGGNTCACANCGCCCGCTGGCGNCGCGTCTCNTCATGCNAGTCNAATGGTNANAGTGGTGCTTGCNCTGATCCTTNAATGGCGGACTGGTGAGTNACGCGTGGGTGACGTACCCTTTGCACGGCGATAGCTGCTANNCNTGNTAAATAACACCTACTACCCTCTATATTTCATANAAGATTTTGAGGAAAGGAAGCTACGGCTTCNCTTAANGATGAGCTTGCNTCCCATTANCTTGTTGGTGAGGTAACGGCCCACCAACGCGACNATGGGTATCCGGCCTGNNAGGGTGAACNGGCACATTGCNNCTGATATACCCCANCTTCTCCTACNGGAGGTCNNNCTANNAATATTCCNCCNTGGACNANANTCNGACGGAGCTNCCCCNCGTGGATGATNAAGGTCCGAANATTGNNNAATCCTTTTATGACTGAANAATAAG TATGGNGAGAGAAACNCCGTATGGNGACNNCANGNCNNGAATAANCANCNGNNNNTTACNNGCCAGCANCCGCGCTNACACGTAAGTTGCGAGCGTNNTTCGGATTATTGNGCGGAAAGGGCNTGNNNGCGGAATTGCAATCTTG | *Spirochaetes* | *Spirochaetes* | *Spirochaetales* | *Spirochaetaceae* | *Treponema* |
| *Treponema vincentii \| HOT-029 \| strain ATCC 35580 \| AF033309 \| Named* | | TTTACGGCTACCTTGTTACGACTTCACCCTCCTTACCAAGCGTACCTTCGGCACCGTCCTCCTTTGCAGGTTAGACAAGCGACTTCGGGTACCCCCAACTCGGATGGTGTGACGGGCGGTGTGTACAAGGCCCGGGAACGTATTCACCNNNNCCGNGCTGATGCGCGATTACTAGCGATTCCAACTTCATGAAGTNNAAGTTTCAGACTTCAATCCGAACTACGATTGCTTTTTTGCGGTT TGCTCCACCTTACGGTCTTGCTTCGCTTTGTAGCAACCATTGTAGCACGTGTGTAGCCCTGGACATAAGGGGCATGATGACTTGACGTCATCCCCGCCTTCCTCCGCGTTCTCCGCGGCAGTCTCCTTTGAGTGCCCAGCTTTACCTGATGGCAACAAAGAACAGGGGTTGCGCTCGTTGCGGGACTTAACCCAACATCTCACGACACGAGCTGACGACAACCATGCACCACCTGTCTCCTCTGTCCGAAGAAATACCCGATTAAGGGTATGTCAGAGGGATGTCAAGTCTTGGTAAGGT TCTTCGCGTTGCTTCGAATTAAACCACATGCTCCGCTGCTTGTGCGGGTCCCCGTCAAT | *Spirochaetes* | *Spirochaetes* | *Spirochaetales* | *Spirochaetaceae* | *Treponema* |
| *Veillonella atypica \| HOT-524 \| strain DSM 20739 \| X84007 \| Named* | | CAATCATCGACTTTACCTTAGACGGCTGGCTCCCGAAGGTTACCCCACCGGCTTTGGGCACTTCCGACTTTCGTGGTGTGACGGGCGGTGTGTACAAGGCCCGGGAACGTATTCACCGCAGTATGCTGACCTGCGATTACTAGCGATTCCGACTTCACGTAGGCGAGTTGCAGCCTACGATCCGAACTGAGAGAGTGTTTCTCGGGTTTGCTCCACCTCGCGGTATTGCTTCCGTCTATTAACTCCCATTGTAGTACGTGTGTAGCCCAGGTCATAAGGGGCATGATGATTTGACGT CATCCCCGCCTTCCTCCGCATTGTCTGCGGCAGTCTCTCATGAGTTCCCACCCGAAGTGCTGGCAACATAAGATAGGGGTTGCGCTCGTTGCGGGACTTAACCCAACATCTCACGACACGAGCTGACGACAACCGTGCACCACCTGTTTTCTGGCTTCCGAAGAAGAGGAACCATCTCTGGTTCTGTCCATCAATGTCAAGACCTGGTAAGGTTCTTCGCGTTGCGTCGAATTAAACCACATACTCCACCGCTTGTGCGGGCCCCCGTCAATTCCT | *Firmicutes* | *Clostridia* | *Clostridiales* | *Veillonellaceae* | *Veillonella* |
| *Veillonella dispar \| HOT-160 \| clone _X031 \| GQ422726 \| Named* | | TTAGAGTTTGATTCTGGCTCAGGACGAACGCTGGTGGCGTGCCTAATACATGCAAGTAGAACGCTGAAGAGAGGAGCTTGCTCTTCTTGGATGAGTTGCGAACGGGTGAGTAACGCGTAGGTAACCTGCCTGATAGCGGGGGATAACTATTGGAAACGATAGCTAATACCGCATGATATTAATTATCGCATGATAATTAATTGAAAGGTGCAATTGCATCACTACCAGATGGACCTGCGTTGTATTAGCTAGTTGGAGGGGTAACGGCCCACCAAGGCGATGATCAGTAGCCGGTCTGAGAGGATGAACGGCCACATTGGGACTGAGACACGGCCCAGACTCCTACGGGAGGCAGCAGTGGGGAATCTTCCGCAATGGACGAAAGTCTGACGGAGCAACGCCGCGTGAGCGATGACGGCCTTCGGGTTGTAAAGCTCTGTTAATCGGGACGAAAGGCCTTCTTGCGAATAGTGAGAAGGATTGACGGTACCGGAATAGAAAGCCACGGCTAACTACGTGCCAGCAGCCGCGGTAATACGTAGGTGGCAAGCGTTGTCCGGAATTATTGGGCGTAAAGCGCGCGCAGGCGGATAGGTCAGT | *Firmicutes* | *Clostridia* | *Clostridiales* | *Veillonellaceae* | *Veillonella* |
| *Veillonella dispar \| HOT-160 \| strain DSM 20735 \| X84006 \| Named* | | TTAGAGTTTGATCATGGCTCAGGACGAACGCTGGCGGCGTGCTTAACACATGCAAGTCGAACGAAGAGCGATGGAAGCTTGCTTCTATCAATCTTAGTGGCGAACGGGTGAGTAACGCGTAATCAACCTGCCCTTCAGAGGGGGACAACAGTTGGAAACGACTGCTAATACCGCATACGATCTAATCTCGGCATCGAGGATAGATGAAAGGTGGCCTCTATTTATAAGCTATCACTGAAGGAGGGGATTGCGTCTGATTAGCTAGTTGGAGGGGTAACGGCCCACCAAGGCGATGATCAGTAGCCGGTCTGAGAGGATGAACGGCCACATTGGGACTGAGACACGGCCCAGACTCCTACGGGAGGCAGCAGTGGGGAATCTTCCGCAATGGACGAAAGTCTGACGGAGCAACGCCGCGTGAGTGATGACGGCCTTCGGGTTGTAAAGCTCTGTTAATCGGGACGAAAGGCCTTCTTGCGAATAGTGAGAAGGATTGACGGTACCGGAATAGAAAGCCACGGCTAACTACGTGCCAGCAGCCGCGGTAATACGTAGGTGGCAAGCGTTGTCCGGAATTATTGGGCGTAAAGCGCGCGCAGG | *Firmicutes* | *Clostridia* | *Clostridiales* | *Veillonellaceae* | *Veillonella* |
| *Veillonella parvula \| HOT-161 \| clone _X002 \| GU350451 \| Named* | | TTAGAGTTTGATCCTGGCTCAGGACGAACGCTGGCGGCGTGCTTAACACATGCAAGTCGAACGAAGAGCGATGGAAGCTTGCTTCTATCAATCTTAGTGGCGAACGGGTGAGTAACGCGTAATCAACCTGCCCTTCAGAGGGGGACAACAGTTGGAAACGACTGCTAATACCGCATACGATCTAATTTCGGCATCGAGGATAGATGAAAGGTGGCCTCTACATGTAAGCTATCACTGAA GGAGGGGATTGCGTCTGATTAGCTAGTTGGAGGGGTAACGGCCCACCAAGGCGATGATCAGTAGCCGGTCTGAGAGGATGAACGGCCACATTGGGACTGAGACACGGCCCAGACTCCTACGGGAGGCAGCAGTGGGGGATCTTCCGCAATGGACGAAAGTCTGACGGAGCAACGCCGCGT GAGTGATGACGGCCTTCGGGTTGTAAAGCTCTGTTAATCGGGACGAAAGGCCTTCTTGCGAATAGTGAGAAGGATTGACGGTACCGGAATAGAAAGCCACGGCTAACTACGTGCCAGCAGCCGCGGTAATACGTAGGTGGCAAGCGTTGTCCGGAATTATTGGGCGTAAAGCGCGCGCAGG | *Firmicutes* | *Clostridia* | *Clostridiales* | *Veillonellaceae* | *Veillonella* |
| *Veillonella parvula \| HOT-161 \| clone BU083 \| AF366266 \| Named* | | TTCGGCTACCTTGTTACGACTTCACCCCAATCATCGACTTTACCTTAGACGGCTGGCTCCCGAAGGTTACCCCACCGGCTTTGGGCACTTCCGACTTTCGTGGTGTGACGGGCGGTGTGTACAAGGCCCGGGAACGTATTCACCGCAGTATGCTGACCTGCGATTACTAGCGATTCCGACTTCACGT AGGCGAGTTGCAGCCTACGATCCGAACTGAGAGAGTGTTTCTCGGGTTTGCTCCATCTCGCGATCTCGCTTCCGTCTATTAACTCCCATTGTAGTACGTGTGTAGCCCAGGTCATAAGGGGCATGATGATTTGACGTCATCCCCGCCTTCCTCCGCATTGTCTGCGGCAGTCTCTCATGAGTTCCCACCATTACGTGCTGGCAACATAAGATAGGGGTTGCGCTCGTTGCGGGACTTAAC CCAACATCTCACGACACGAGCTGACGACAACCGTGCACCACCTGTTTTCTGGCTTCCGAAGAAGAGGAACTATCTCTAGTTCTGTCCATCAATGTCAAGACCTGGTAAGGTTCTTCGCGTTGCGTCGAATTAAACCACATACTCCACCGCTTGTGCGGGCCCCCGTCAATTCC | *Firmicutes* | *Clostridia* | *Clostridiales* | *Veillonellaceae* | *Veillonella* |
| *Veillonella parvula \| HOT-161 \| strain DSM 2008 \| X84005 \| Named* | | TTTACGGTTACCTTGTTACGACTTCACCCCAATCATCGACTTTACCTTAGACGGC TGGCTCCCGAAGGTTACCCCACCGGCTTTGGGCACTTCCGACTTTCGTGGTGTGACGGGCGGTGTGTACAAGGCCCGGGAACGTATTCACCGCAGTATGCTGACCTGCGATTACTAGCGATTCCGACTTCACGTAGGCGAGTTGCAGCCTACGATCCGAACTGAGAGAGTGTTTCTCGGGTTTGCTCCATCTCGCGATCTCGCTTCCGTCTATTAACTCCCATTGTAGTACGTGTGTAGCCCAGGTCATAAGGGGCATGATGATTTGACGTCATCCCCGCCTTCCTCCGCATTATCTGCGGCAGTCTCTCATGAGTTCCCACCCAAAGTGCTGGCAACATAAGATAGGGGTTGCGCTCGTTGCGGGACTTAACCCAACATCTCACGACACGAGCTGACGACAACCGTGCACCACCTGTTTTCTGGCTTCCGAAGAAGAGGAACCATCTCTGGTTCTGTCCATCAATGTCAAGACCTGGTAAGGTTCTTCGCGTTGCGTCGAATTAAACCACATACTCCACCGCTTGTGCGGGCCCCCGTCAATTC | *Firmicutes* | *Clostridia* | *Clostridiales* | *Veillonellaceae* | *Veillonella* |
| *Veillonellaceae [G-1] sp. \| HOT-132 \| clone CS015 \| AF287791 \| Phylotype* | | TTAGAGTTTGATCCTGGCTCAGGACGAACGCTGGCGGCGTGCTTAACACATGCAAGTCGAACGGAGCTGTTTATTTCGGTAGATAGCTTAGTGGCAAACGGGTGAGTAACGCGTAGGCAACCTGCCCTTAGGATGGGGACAACGGCCCGAAAGGACCGCTAATACCGAATGGACTCTAACTTTCGCATGGAAGAAAGAGGAAAGATGGTGCAAGCCATCGCCGAAGGAAGGGCCTGCGTCTGATTAGCCAGTTGGTGAGGTAACGGCTCACCAAAGCGACGATCAGTAGCCGGTCTGAGAGGATGAACGGCCACAATGGGACTGAGACACGGCCCAGACTCCTACGGGAGGCAGCAGTGGGGAATCTTCCGCAATGGACGAAAGTCTGACGGAGCAACGCCGCGTGAGTGATGACGGCCTTCGGGTTGTAAAGCTCTGTTAATCGGGACGAAAGGCCTTCTTGCGAATAGTGAGAAGGATTGACGGTACCGGAATAGAAAGCCACGGCTAACTACGTGCCAGCAGCCGCGGTAATACGTAGGTGGCAAGCGTTGTCCGGAATTATTGGGCGTAAAGCGCGCGCAGGCGGATAGGTCAGTC | *Firmicutes* | *Clostridia* | *Clostridiales* | *Veillonellaceae* | *Veillonellaceae[G-1]* |
| ***Coronary balloon*** | | | | | | | |
| **Phylotype** | **Sequence** | | **Phylum** | **Class** | **Order** | **Family** | **Genus** |
| *Actinomyces sp. \| HOT-178 \| strain B27SC \| AF287750 \| Unnamed* | CGGCGGGGCCGANCCNAGGGCCTCACTAAACCATCCAATCGGTAGTAGCGACGGGCGGTGTGTACAAAGGGCAGGGACTTAATCAACGCAAGCTTATGACCCGCACTTACTGGGAATTCCTCGTTCATGGGGAANAATTGCAATCCCCGATCCCCATCACGAATGGGGTTCAACGGGTTACCCGCGCCTGCCGGCGTAGGGTAGGCACACGCTGAGCCAGGATCAAACTCTAAGGGCGAATTCCAGCACACTGGCGGCCGTTACTAGTGGATCCGAGCTCGGTACCAAGCTTGGCGTAATCATGGTCATAGCTGTTTCCTGTGTGAAATTGTTATCCGCTCACAATTCCACACAACATACGAGCCGGAAGCATAAAGTGTAAAGCCTGGGGTGCCTAATGAGTGAGCTAACTCACATTAATTGCGTTGCGCTCACTGCCCGCTTTCCAGTCGGGAAACCTGTCGTGCCAGCTGCATTAATGAATCGGCCAACGCGCGGGGAGAGGCGGTTTGCGTATTGGGCGCTCTTCCGCTTCCTCGCTCACTGACTCGCTGCGCTCGGTCGTTCGGCTGCGGCGAGCGGNATCAGCTCACTCAAAGG | | *Actinobacteria* | *Actinobacteria* | *Actinomycetales* | *Actinomycetaceae* | *Actinomyces* |
| *Achromobacter xylosoxidans \| HOT-343 \| strain DSM 30026 \| Y14907 \| Named* | TTTACGGCTACCTTGTTACGACTTCACCCCAGTCACGAACCCTGCCGTGGTAAGCGCCCTCCTTGCGGTTAAGCTACCTACTTCTGGCAGAACCCGCTCCCATGGTGTGACGGGCGGTGTGTACAAGACCCGGGAACGTATTCACCGCGACATGCTGATCCGCGATTACTAGCGATTCCAACTTCATGTAGTCGAGTTGCAGACTACAATCCGGACTACGATACACTTTCTGGGATTAGCTCCCCCTCGCGGGTTGGCGGCCCTCTGTATGTACCATTGTATGACGTGTGAAGCCCTACCCATAAGGGCC ATGAGGACTTGACGTCATCCCCACCTTCCTCCGGTTTGTCACCGGCAGTCTCATTAGAGTGCCCTTTCGTAGCAACTAATGACAAGGGTTGCGCTCGTTGCGGGACTTAACCCAACATCTCACGACACGAGCTGACGACAGCCATGCAGCACCTGTGTGCAGGTTCTCTTGCGAGCACTC CCAAATCTCTTCGGGATTCCTGCCATGTCAAGGGTAGGTAAGGTTTTTCGCGTTGCATCGAATTAATCCACATCATCCACCGCTTGTGCGGGTCCCCGTCAATTCCTTTG | | *Proteobacteria* | *Betaproteobacteria* | *Burkholderiales* | *Alcaligenaceae* | *Achromobacter* |
| *Acinetobacter baumannii \| HOT-554 \| strain DSM 30007 \| X81660 \| Named* | TTAGAGTTTGATCATGGCTCAGATTGAACGCTGGCGGCAGGCTTAACACATGCAAGTCGAGCGGGGAAGGGTAGCTTGCTACCTGACCTAGCGGCGGACGGGTGAGTAATGCTTAGGAATCTGCCTATTAGTGGGGGACAACATTCCGAAAGGAATGCTAATACCGCATACGCCCTACGGGGGAAAGCAGGGGATCTTCGGACCTTGCGCTAATAGATGAGCCTAAGTCAGATTAGCTAGTTGGTGGGGTAAAGGCCTACCAAGGCGACGATCTGTAGCGGGTCTGAGAGGATGATCCGCCACACTGGGACTGAGACACGGCCCAGACTCCTACGGGAGGCAGCAGTGGGGAATATTGGACAATGGGCGCAAGCCTGATCCAGCCATGCCGCGTGTGTGAAGAAGACCTTTTGGTTGTAAAGCACTTTAAGCGAGGAGGAGGCTACTTGGATTAATACTCTAGGATAGTGGACGTTACTCGCAGAATAAGCACCGGCTAACTCTGTGCCAGCAGCCGCGGTAATACAGAGGGTGCGAGCGTTAATCGGATTTACTGGGCGTAAAGCGTGCGTAGGCGGCTTCTTAANTCGGATGTGAAAT | | *Proteobacteria* | *Gammaproteobacteria* | *Pseudomonadales* | *Moraxellaceae* | *Acinetobacter* |
| *Acinetobacter sp. \| HOT-408 \| clone C4AKM094 \| AY278636 \| Phylotype* | TTAGAGTTTGATCATGGCTCAGATTGAACGCTGGCGGCAGGCTTAACACATGCAGGTCGAGCGGGGTGATGTAGCTTGCTACATTACCTAGCGGCGGACGGGTGAGTAATGCTTAGGAATCTGCCTATTAGTGGGGGACAACATTCCGAAAGGAATGCTAATACCGCATACGCCCTACGGGGGAAAGCAGGGGATCTTCGGACCTTGCGCTAATAGATGAGCCTAAGTCAGATTAGCTAGTTGGTGGGGTAAAGGCCTACCAAGGCGACGATCTGTAGCGGGTCTGAGAGGATGATCCGCCACACTGGGACTGAGACACGGCCCAGACTCCTACGGGAGGCAGCAGTGGGGAATATTGGACAATGGGCGAAAGCCTGATCCAGCCATGCCGCGTGTGTGAAGAAGGCCTTTTGGTTGTAAAGCACTTTAAGCGAGGAGGAGGCTACTTAGATTAATACTCTAGGATAGTGGACGTTACTCGCAGAATAAGCACCGGCTAACTCTGTGCCAGCAGCCGCGGTAATACAGAGGGTGCGAGCGTTAATCGGATTTACTGGGCGTAAAGCGTGCGTAGGCGGCTTTTTAAGNCGGATGTGAAAT | | *Proteobacteria* | *Gammaproteobacteria* | *Pseudomonadales* | *Moraxellaceae* | *Acinetobacter* |
| *Actinomyces sp. \| HOT-525 \| clone MB6_C03 \| DQ003632 \| Phylotype* | TTTACGGTTACCTTGTTACGACTTCGTCCCAATCACCGGCCCCGCCTTCGACCGCTCCCCATGGGGCCACGGGCTTCGGGCGTCGCCGACTTTCATGACGTGACGGGCGGTGTGTACAAGGCCCGAGAACGTATTCACCGCAGCAGTGCTGATCTGCGATTACTAGCGACTCCGACTTCACGGTGTCGAGTTGCAGACACCGATCCGAACTGAGACCGGCTTTAAGGGATTCGCCCCGCCTCGCGGCATCGCAACCCTCTGTACCGGCCATTGTAGCATGCGTGAAGCCCAGGACGTCAGGGGCATGATGATTTGACGTCGTCCCCACCCTCCTCCGAGTTGACCCCGGCAGTCTCCCGAGAGTCCCCGCCACGACGCGCTGGCAACACGGGACAGGGGTTGCGCTCGTTGCGGGACTTAA CCCAACATCTCACGACACGAGCTGACGACAACCATGCACCACCTGCGGGGGCGCCCCGAAAAAGGGAGACGCCGTCTCCGACGCCACCGCCCCCATGTCAAGCCCTGGTAAGGTTCTTCGCGTTGCATCGAATTAATCCGCATGCTCCGCCGCTTGTGCGGGCCCCCGTCAATTCCTTT | | *Actinobacteria* | *Actinobacteria* | *Actinomycetales* | *Actinomycetaceae* | *Actinomyces* |
| *Alloprevotella tannerae \| HOT-466 \| strain ATCC 51259 \| AJ005634 \| Named* | TTTACGGTTACCTTGTTACGACTTAGCCCCAATCACCAGTTTTGCCCTAGGCCGATCCTTGCGGTCACGGACTTCAGGCACCCCCGGCTTTCATGGCTTGACGGGCGGTGTGTACAAGGCCCGGGAACGTATTCACCGCGCCATGGCTGATGCGCGATTACTAGCGAATCCGGCTTCGTGGAGTCGGGTTGCAGACTCCAGTCCGAACTGAGGCCGGGTTTCGGGATCAGCATCCTGTCGCC AGGTAGCTTCCTGCTGCTCCGACCATTGTAACACGTGTGTAGCCCCGGACGTAAGGGCCGTGCTGATTTGACGTCATCCCCACCTTCCTCCGCCTTGCGGCGGCAGTATCCACGGAGTTCCCGGCATTACCCGATGGCAAACGTAGAAAAGGGTTGCGCTCGTTATGGCACTTAAGCCGACACCTCACGGCACGAGCTGACGACAACCATGCAGCACCTCCAAAAGTGTCCGAAGAAAAAGCGCATCTCTGCACTCCGCACTCTTGGTTCAAGCCCGGGTAAGGTTCCTCGCGTATCATCGAATTAAACCACATGTTCCTCCGCTTGTGCGGGCCCCCGTCAATTCCTTTGAGTTTCA | | *Bacteroidetes* | *Bacteroides* | *Bacteroidales* | *Prevotellaceae* | *Alloprevotella* |
| *Atopobium rimae \| HOT-750 \| strain ATCC49626 \| AF292371 \| Named* | TTAGAGTTTGATCATGGCTCAGGATGAACGCTGGCGGCGCGCCTAACACATGCAAGTCGAAC GGTTAAAGCACCTTTTAGGTGTGTATAAAGTGGCGAACGGCTGAGTAACACGTGGGCAACCTGCCCTCCTCTTGGGGATAGCCTCGGGAAACCGAGGATAATACCCGATACTTCAATGTGGCCGCATGACGACATTGAGAAAGCTTTTGCGGAGAAGGATGGGCCCGCGGCCTGTTAGCTTGTTGGTGGGGTAGAGGCCTACCAAGGCAATGATGGGTAGCTGGGTTGAGAGACCGACCAGCCAGATTGGGACTGAGACACGGCCCAGACTCCTACGGGAGGCAGCAGTGGGGAATCTTGCACAATGGGCGAAAGCCTGATGCAGCGACGCCGCGTGCGGGAAGAAGGCCTTCGGGTTGTAAACCGCTTTCAGCAGGGACGAGGCGATAAGTGACGGTACCTGCAGAAGAAGCCCCGGCTAACTACGTGCCAGCAGCCGCGGTAATACGTAGGGGGCAAGCGTTATCCGGATTCATTGGGCGTAAAGCGCTCGTAGGCGGTCTGTTAGGTCGGGAGTTAAATCCGGAGGCTCAACCT | | *Actinobacteria* | *Actinobacteria* | *Coriobacteriales* | *Coriobacteriaceae* | *Atopobium* |
| *Bartonella sp. \| HOT-001 \| strain A28SC \| GQ422708 \| Unnamed* | TTCGGTTACCTTGTTACGACTTCACCCCAGTCGCTGACCCTACCGTGGTCGCCTGCCTCCTTGCGGTTGGCGCAGCGCCGTCGGGTAAGACCAACTCCCATGGTGTGACGGGCGGTGTGTACAAGGCCCGGGAACGTATTCACCGTGGCGTGCTGATCCACGATTACTAGCGATTCCGCCTTCATGCACCCGAGTTGCAGAGTGCAATCCGAACT GAGACGGTTTTTGGGGATTTGCTCCACCTCGCGGCTTCGCGTCCCACTGTCACCGCCATTGTAGCACGTGTGTAGCCCATCCCGTAAGGGCCATGAGGACTTGACGTCATCCACACCTTCCTCGCGGCTTATCACCGGCAGTCTCCCTAGAGTGCCCAACTGAATGATGGCAACTAAGGACGTGGGTTGCGCTCGTTGCGGGACTTAACCCAACATCTCACGACACGAGCTGACGACAGCCATGCAGCACCTGTGTGCACGCCTCCGAAGAGGATCCCCGATCTCTCGAGGTAACATGCCATGTCAAGGGATGGTAAGGTTCTGCGCGTTGCTTCGAATTAAACCACATGCTCCACCGCTTGTGCGGGCCCCCGTCAATTCCTTT | | *Proteobacteria* | *Alphaproteobacteria* | *Rhizobiales* | *Bartonellaceae* | *Bartonella* |
| *Burkholderia cepacia \| HOT-571 \| strain ATCC 25416 \| AF097530 \| Named* | TTAGAGTTTGATCCTGGCTCAGATTGAACGCTGGCGGCATGCCTTACACATGCAAGTCGAACGGCAGCACGGGTGCTTGCACCTGGTGGCGAGTGGCGAACGGGTGAGTAATACATCGGAACATGTCCTGTAGTGGGGGATAGCCCGGCGAAAGCCGGATTAATACCGCATACGATCTACGGATGAAAGCGGGGGACCTTCGGGCCTCGCGCTATAGGGTTGGCCGATGGCTGATTAGCTAGTTGGTGGGGTAAAGGCCTACCAAGGCGACGATCAGTAGCTGGTCTGAGAGGACGACCAGCCACACTGGGACTGAGACACGGCCCAGACTCCTACGGGAGGCAGCAGTGGGGAATTTTGGACAATGGGCGAAAGCCTGATCCAGCAATGCCGCGTGTGTGAAGAAGGCCTTCGGGTTGTAAAGCACTTTTGTCCGGAAAGAAATCCTTGGCTCTAATACAGTCGGGGGATGACGGTACCGGAAGAATAAGCACCGGCTAACTACGTGCCAGCAGCCGCGGTAATACGTAGGGTGCGAGCGTTAATCGGAATTACTGGGCGTAAAGCGTGCGCAGGCGGTTTGCTAAGACCGATGTGAAA | | *Proteobacteria* | *Betaproteobacteria* | *Burkholderiales* | *Burkholderiaceae* | *Burkholderia* |
| *Capnocytophaga leadbetteri \| HOT-329 \| clone BR085 \| GU350453 \| Named* | TTTACGGTTACCTTGTTACGACTTAGCCCCAGTCACTAGTTTTACCCTAAACAGCTCCTCGCGGTGACCGTCTTCAGGTACCCCCAGCTTCCATGGCTTGACGGGCGGTGTGTACAAGGCCCGGGAACGTATTCACCGGATCATGGCTGATATCCGATTACTAGCGATTCCAGCTTCACGGAGTNCGAGTTGCAGACTCCGATCCGAACTGTGATCGTCTTTATAGATTCGCGCCTGCTCACGCAGTGGCTGCTCTCTGTAACGACCATTGTAGCACGTGTGTAGCCCAAGATGTAAGGGCCGTGATGATTTGACGTCATCCCCACCTTCCTCACGGTTTGCACCGGCAGTCCCACTAGAGTGCTCGACTCGACTCGCTAGCAACTAATGGCAGGGGTTGCGCTCGTTATAGGACTTAACCTGACACCTCACGGCACGAGCTGACGACAACCATGCAGCACCTTGAAAACTGTCCGAAGAAAANCGTATCTCNACACCTGTCNNTCTCCATTTAAACCTTGGTAANNNTNCTCGCGTATCANCNAATTAAANCACATGCTCCANCGCTTGTGCGGGCCCCCNTCNATTCCTTTGAGTTTC | | *Bacteroidetes* | *Flavobacteria* | *Flavobacteriales* | *Flavobacteriaceae* | *Capnocytophaga* |
| *Enterobacter cancerogenus \| HOT-565 \| strain LMG 2693 \| Z96078 \| Named* | TTTACGGTTACCTTGTTACGACTTCACCCCAGTCATGAATCACAAAGTGGTAAGCGCCCTCCCGAAGGTTAAGCTACCTACTTCTTTTGCAACCCACTCCCATGGTGTGACGGGCGGTGTGTACAAGGCCCGGGAACGTATTCACCGTAGCATTCTGATCTACGATTACTAGCGATTCCGACTTCATGGAGTCGAGTTGCAGACTCCAATCCGGACTACGACGCACTTTATGAGGTCCGCTTGCTCTCGCGAGGTCGCTTCTCTTTGTATGCGCCATTGTAGCACGTGTGTAGCCCTACTCGTAAGGGCCATGATGACTTGACGTCATCCCCACCTTCCTCCAGTTTATCACTGGCAGTCTCCTTTGAGTTCCCGGCCTAACCGCTGGCAACAAAGGATAAGGGTTGCGCTCGTTGCGGGACTTAACCCAACATTTCACAACACGAGCTGACGACAGCCATGCAGCACCTGTCTCAGAGTTCCCGAAGGCACCAAAGCATCTCTGCTAAGTTCTCTGGATGTCAAGAGTAGGTAAGGTTCTTCGCGTTGCATCGAATTAAACCACATGCTCCACCGCTTGTGCGGGCCCCCGTCAATTCA | | *Proteobacteria* | *Gammaproteobacteria* | *Enterobacteriales* | *Enterobacteriaceae* | *Enterobacter* |
| *Enterobacter hormaechei \| HOT-634 \| strain DSMZ 16691 \| AJ853890 \| Named* | TTAGAGTTTGATCATGGCTCAGATTGAACGCTGGCGGCAGGCCTAACACATGCAAGTCGAGCGGCAGCGGAAAGTAGCTTGCTACTTTGCCGGCGAGCGGCGGACGGGTGAGTAATGTCTGGGAAACTGCCTGATGGAGGGGGATAACTACTGGAAACGGTA GCTAATACCGCATAACGTCGCAAGACCAAAGAGGGGGACCTTCGGGCCTCTTGCCATCAGATGTGCCCAGATGGGATTAGCTAGTAGGTGGGGTAACGGCTCACCTAGGCGACGATCCCTAGCTGGTCTGAGAGGATGACCAGCCACACTGGAACTGAGACACGGTCCAGACTCCTACGGGAGGCAGCAGTGGGGAATATTGCACAATGGGCGCAAGCCTGATGCAGCCATGCCGCGTGTATGAAGAAGGCCTTCGGGTTGTAAAGTACTTTCAGCGGGGAGGAAGGTGTTGAGGTTAATAACCTCAGCAATTGACGTTACCCGCAGAAGAAGCACCGGCTAACTCCGTGCCAGCAGCCGCGGTAATACGGAGGGTGCAAGCGTTAATCGGAATTACTGGGCGTAAAGCGCACGCAGGCGGTCTGTCAAGTCGGANGT | | *Proteobacteria* | *Gammaproteobacteria* | *Enterobacteriales* | *Enterobacteriaceae* | *Enterobacter* |
| *Fusobacterium nucleatum ss vincentii \| HOT-200 \| strain ATCC 49256 \| NZ_AABF02000026 \| Named* | TTAGAGTTTGATCCTGGCTCAGGATGAACGCTGACAGAATGCTTAACACATGCAAGTCAACTT GAATTTGGGTTTTTAACTTAGGTTTGGGTGGCGGACGGGTGAGTAACGCGTAAAGAACTTGCCTCACAGCTAGGGACAACATTTGGAAACGAATGCTAATACCTAATATTATGATAATAGGGCATCCTATAATTATGAAAGCTATAAGCGCTGTGAGAGAGCTTTGCGTCCCATTAGCTAGTTGGAGAGGTAACGGCTCACCAAGGCGATGATGGGTAGCCGGCCTGAGAGGGTGATCGGCCACAAGGGGACTGAGACACGGACCTTACTCCTACGGGAGGCAGCAGTGGGGAATATTGGACAATGGACCAAGAGTCTGATCCAGCAATTCTGTGTGCACGATGAAGTTTTTCGGAATGTAAAGTGCTTTCAGTTGGGAAGAAAGAAATGACGGTACCAACAGAAGAAGTGACGGCTAAATACGTGCCAGCAGCCGCGGTAATACGTATGTCACGAGCGTTATCCGGATTTATTGGGCGTAAAGCGCGTCTAGGTGGTTATATAAGTCTGATGTGAAAATGCAGGGCTCAACTCTGT | | *Fusobacteria* | *Fusobacteria* | *Fusobacteriales* | *Fusobacteriaceae* | *Fusobacterium* |
| *Fusobacterium nucleatum ss. nucleatum \| HOT-698 \| strain ATCC 25586 \| AJ133496 \| Named* | TTAGAGTTTGATCATGGCTCAGGATGAACGCTGACAGAATGCTTAACACATGCAAGTCTACTTGAATTTGGGTTTTTTAACTTCGATTTGGGTGGCGGACGGGTGAGTAACGCGTAAAGAACTTGCC TCACAGCTAGGGACAACATTTGGAAACGAATGCTAATACCTAATATTATGATTATAGGGCATCCTAGAATTATGAAAGCTATATGCGCTGTGAGAGAGCTTTGCGTCCCATTAGCTAGTTGGAGAGGTAACGGCTCACCAAGGCAATGATGGGTAGCCGGCCTGAGAGGGTGAACGGCCACAAGGGGACTGAGACACGGCCCTTACTCCTACGGGAGGCAGCAGTGGGGAATATTGGACAATGGACCGAGAGTCTGATCCAGCAATTCTGTGTGCACGATGACGTTTTTCGGAATGTAAAGTGCTTTCAGTTGGGAAGAAAAAAATGACGGTACCAACAGAAGAAGTGACGGCTAAATACGTGCCAGCAGCCGCGGTAATACGTATGTCACGAGCGTTATCCGGATTTATTGGGCGTAAAGCGCGTCTAGGTGGTTATGTAAGTCTGATGTGAAAATGCAGGGCTCAACTCTG | | *Fusobacteria* | *Fusobacteria* | *Fusobacteriales* | *Fusobacteriaceae* | *Fusobacterium* |
| *GN02 [G-1] sp. \| HOT-872 \| clone CN02 \| JX294353 \| Phylotype* | TTAGAGTTTGATCATGGCTCAGGGTGAACGCTAGCGGTGCGCCTAACACATGCAAGTCGAGCGGGAGTTGCACAGAGGAGACTTCGGTTGAATCGGTGTAATGATAGCGGCGAACGGGTGAGTAACACGTTGGAACCTACCCCCAAGTCAGGGATAGCCAAGGGAAACCTTGGATAATACCGGATAGTCTCTTAGGAGTAAAAATTTATTGCTTGGGGAGGGGCCTGCGGACTATCAGCTAGTTGGTAAGGTAAGAGCTTACCAAGGCTATGACGGATAACTGGTCTGAGAGGATGATCAGTCACAATGGAACTGAGACACGGTCCATACTCCTACGGGAGGCAGCAGTGAGGAATCTTCCACAATGGACGAAAGTCTGATGGAGCGACACCGCGTGAAGGAAGAAGGCCTAACGGTTGTAAACTTCTTTTCTGAAGGAGCATAATGAGAGTACTTTAGGAATAAGGGACGGCTAAATACGTGCCAGCAGCCGCGGTAATACGTATGTCTCGAGCGTTACCCGGAATAACTGGGTGTAAAGGGTTTGTAGGTTGATGGTTAAGTCAGATATGAAAGACCGAAGCTTAACTTCGAGTTAGA | | *GN02* | *GN02[C-1]* | *GN02[O-1]* | *GN02[F-1]* | *GN02[G-1]* |
| *Haemophilus haemolyticus \| HOT-851 \| strain F0397 \| HM596277 \| Named* | TTTACGGTTACCTTGTTACGACTTGATCCTGAGCCATGATCAAACTCTAAGGGCGAATTCTGCAGATATCCATCACACTGGCGGCCGCTCGAGCATGCATCTAGAGGGCCCAATTCGCCCTATAGTGAGTCGTATTACAATTCACTGGCCGTCGTTTTACAACGTCGTGACTGGGAAAACCCTGGCGTTACCCAACTTAATCGCCTTGCAGCACATCCCCCTTTCGCCAGCTGGCGTAATAGCGAAGAGGCCCGCACCGATCGCCCTTCCCAACAGTTGCGCAGCCTGAATGGCGAATGGACGCGCCCTGTAGCGGCGCATTAANCGCGGCGGGTGTGGTGGTTACNCGCAGCGTGACCGCTACACTTGCCAGCGCCCTANCGCCCGCTCCTTTCGCTTTCTTCCGTTCCTNTCTCGCCAC | | *Proteobacteria* | *Gammaproteobacteria* | *Pasteurellales* | *Pasteurellaceae* | *Haemophilus* |
| *Moraxella osloensis \| HOT-711 \| strain Ben 58 \| X95304 \| Named* | TTTACGGCTACCTTGTTACGACTTCACCCCAGTCATCGACCACACCGTGGTAAGCGCCCTCTTACGTTAGGCTACCTACTTCTGGTGCAATAGACTCCCATGGTGTGACGGGCGGTGTGTACAAGGCCCGGGAACGTATTCACCGCAGCATTCTGATCTGCGATTACTAGCGATTCCGACTTCATGGAGTCGAGTTGCAGACTCCAATCCGGACTACGATAGGCTTTTTGAGATTAGCATCACATCGCTGTGTAGCAACCCTCTGTACCTACCATTGTAGCACGTGTGTAGCCCTGGTCGTAAGGGCCATGATGACTTGACGTCGTCCCCGCCTTCCTCCAGTTTGTCACTGGCAGTATCCTTAAAGTTCCCGGCTTAACCCGCTGGCAAATAAGGAAAAGGGTTGCGCTCGTTGCGGGACTTAACCCAACATCTCACGACACGAGCTGACGACAGCCATGCAGCACCTGTATGTGAATTCCCGAAGGCACTCCCGCATCTCTGCAGGATTCTCACTATGTCAAGACCAGGTAAGGTTCTTCGCGTTGCATCGAATTAAACCACATGCTCCACCGCTTGTGCGGGCCCCCGTCAATTCATTTGAGTTTTAACCTT GCGGCCGTACTCCCCAGGCGGTCTACTTATCGCGTTAACT | | *Proteobacteria* | *Gammaproteobacteria* | *Pseudomonadales* | *Moraxellaceae* | *Moraxella* |
| *Neisseria sicca \| HOT-764 \| strain Q13 \| AJ239292 \| Named* | TTTACGGTTANNNNANNNNNNNNCNCCCCAGTCATGAAGCATACCGTGGTAANNNNNNNNNNNNNNGTTACCCTACCTACTTCTGGTATCCCCCACTCCCATGGTGTGACGGGCGGTGTGTACAA GACCCGGGAACGTATTCACCGCAGTATGCTGACCTGCGATTACTAGCGATTCCNACTTCATGCACTCGAGTTACAGAGTGCAATCCGGACTACGATCGGTTTTGTGAGATTGGCTCCACCTCGCGGCTTGGCTACCCTCTGTACCGACCATTGTATGACGTGTGAAGCCCTGGTCATAAGGGCCATGAGGACTTGACGTCATCCCCACCTTCCTCCGGCTTGTCACCGGCAGTCTCATTAGAGTGCCCAACTTAATGATGGCAACTAATGACAAGGGTTGCNCTCGTTGCGGGACTTAACCCAACATCTCACGACACGAGCTGACGACAGCCATGCAGCACCTGTGTTACGGCTCCCGAA GGCACCCCTCCGTCTCTGGAGGGTTCCGTACATGTCAAGACCAGGNAANGTTCTTCGCGTTGCATCNNATTAATCCACATCATCCACCGCTTGTGCGGGTCCCCGTCANTTCCTT | | *Proteobacteria* | *Betaproteobacteria* | *Neisseriales* | *Neisseriaceae* | *Neisseria* |
| *Neisseria subflava \| HOT-476 \| strain U37 \| AJ239291 \| Named* | TTTACGGTTACCTTGTTACGACTTCACCCCAGNCATGAAGCATACCGTGGTAAGCGGGCTCCTTGCGGTTACCCTACCTACTTCTGGTATCCCCCACTCCCATGGTGTGACNGGCGGTGTGTACAAGACCCGGGAACGTATTCACCGCAGTATGCTGACCTGCGATTACTAGCGATTCCGACTTCATGCACTCGAGTTGCAGAGTGCAATCCGGACTACGATCGGTTTTGTGAGATTGGCTCCACCTCGCGGCTTGGCTACCCTCTGTACCGACCATTGTATGACGTGTGAAGCCCTGGTCATAAGGGCCATGAGGACTTGACGTCATCCCCACCTTCCTCCGGCTTGTCACCGGCAGTCTCATTAGAGTGCCCAACTAAATGATGGCAACTAATGACAAGGGTTGCGCTCGTTGCGGGACTTAACCCAACATCTCACGACACGAGCTGACGACAGCCATGCAGCACCTGTGTTACGGCTCCCGAAGGCACTCCTCCGTCTCTGGAGGATTCCGTACATGTCAAGACCAGGTAAGGTTCTTCGCGTTGCATCGAATTAATCCACATCATCCACCGCTTGTGCGGGTCCCCGTCAATTCCT | | *Proteobacteria* | *Betaproteobacteria* | *Neisseriales* | *Neisseriaceae* | *Neisseria* |
| *Parvimonas sp \| HOT-110 \| clone HE064 \| GQ422714 \| Unnamed* | TTTACGGTTACCTTGTTACGACTTCACCCCAGTCATNAATCCTACCTTCGACTGCTCCTCAATTAGGTCACAGGCTTCGGGTATTATCAACTCCCATGGTGTGACGGGCGGTGTGTACAAGACCCGGGAACGCATTCACCGCGACATTCTGATCCGCGATTACTAGCAACTCCGACTTCATGTAGGCGAGTTGCAGCCTACAATCCGAACTGGGATTGGCTTTGGAGTTTTGCATTATATCACTATATAGCTTCCCTCTGTACCAACCATTGTAGCACGTGTGTAGCCCAGGACATAAAGGGCATGATGATTTGACGTCATCCCCACCTTCCTCCGATTTGTCATCGGCAGTCTACTTAGAGTCCCCGGCATTATCCGCTGGTAACTAAGTATAGGGGTTGCGCTCGTTGCGGGACTTAACCCAACATCTCACGACACGAGCTGACGACAACCATGCACCACCTGTATGGATGTCTNATAAAGAGAGGGGTATATCTCTATACCTTTCACCCACATGTCAAGCCCTGGTAAGGTTCTTCGCGTTGCATCNAATTAAACCACATGCTCCGCTGC TTGTGCGGGTCCCCGTCAATTCCTTTG | | *Firmicutes* | *Clostridia* | *Clostridiales* | *Peptostreptococcaceae[13]* | *Parvimonas* |
| *Peptostreptococcus stomatis \| HOT-112 \| strain A21H2 \| GQ422715 \| Named* | NNGNTGTGACGGGCGGTGTGTACAAGACCCGGGAACGCATTCACCGCAGCATTCTGATCTGCNATTACTAGTAACTCCAGCTTCATGTAGGCGAGTTTCAGCCTACAATCCGAACTGAGAATGGCTTTAAGGGATTGGCTCCACCTCGCGGTTTGGCAACCCTCTGTACCACCCATTGTAGCACGTGTGTAGCCCTAAGCATAAGGGGCATGATGATTTGACGTCATCCCCACCTTCCTCCAGGTTATCCCTGGCAGTCTCTCTAGAGTGCCCAACTGAATGCTGGCAACTAAAGACAAGGGTTGCGCTCGTTGCGGGACTTAACCCAACATCTCACGACACGAGCTGACGACAACCATGCACCACCTGTCACCTCAGTCCCGAAGGAAGGGTGTGATTAAACACCTGTCCGAGGGATGTCAAGCTTAGGTAAGGTTCTTCGCGTTGCTTCGAATTAAACCACATGCTCCGCTACTTGTGCGGGTCCCCGTCAATTCCTTTGAGTTTCACACTTGCGTGCGTACTCCCCAGGCGGAGTACTTAATGCGTTAGCTGCGGCACCGAGGGGGGTAACCCCCGACACCTAGTACTCATCGTTTA | | *Firmicutes* | *Clostridia* | *Clostridiales* | *Peptostreptococcaceae[11]* | *Peptostreptococcus* |
| *Porphyromonas endodontalis \| HOT-273 \| clone AJ002 \| AY005067 \| Named* | TTTACGGTTACCTTGTTACGACTTAGCCCCAGTCACTGGTATTACCCTTAAGCGCCCCTTGCGGTTACGCTCTTCAGGTACTCCCAACTTCCATGGCTTGACGGGCGGCGTGTACAAGGCCCGGGAACGTATTCACCGCGCCATGGCTGATGCGCGATTACTAGCGAATCCAGCTTCACGGAGTCGAGTTGCAGACTCCGATCCGAACTGGGACAGGGTTTGGAGATCCGCTTC ATGTCACCATGTCGCTTCCCTTTGTCCCTGCCATTGTAACACGTGTGTCGCCCCGGATGTAAGGGCCGTGCTGATTTGACGTCATCCGCCCCTTCCTCTCGTCTTACGACGGCTGTCTCGATAGAGTCCTCAGCATGACCTGTTAGTAACTATCGACGCGGGTTGCGCTCGTTATGGCACTTAAGCCGACACCTCACGGCACGAGCTGACGACAACCATGCAGCACCTACTTAGATGTCCCGAAGGAAAGCAAACTCTCATCTGCCACCATCTAAATTTCAATCCCGGGTAAGGTTCCTCGCGTATCATCGAATTAAACC ACATGTTCCTCCGCTTGTGCGGGCCCCCGTCAATTCCTTTGAGTTT | | *Bacteroidetes* | *Bacteroides* | *Bacteroidales* | *Porphyromonadaceae* | *Porphyromonas* |
| *Porphyromonas gingivalis \| HOT-619 \| strain DSM 20709 \| X73964 \| Named* | TTAGAGTTTGATCCTGGCTCAGGATGAACGCTAGCGATAGGCTTAACACATGCAAGTCGAGGGGCAGCATGATCTTAGCTTGCTAAGGTTGATGGCGACCGGCGCACGGGTGCGTAACGCGTATGCAACTTGCCTTACAGAGGGGGATAACCCGTTGAAAGACGGACTAAAACCGCATACACTTGTATTATTGCATGATATTACAAGGAAATATTTATAGCTGTAAGATAGGCATGCGTCCCATTAGCTAGTTGGTGAGGTAACGGCTCACCAAGGCGACGATGGGTAGGGGAACTGAGAGGTTTATCCCCCACACTGGTACTGAGACACGGACCAGACTCCTACGGGAGGCAGCAGTGAGGAATATTGGTCAATGG GCGAGAGCCTGAACCAGCCAAGTCGCGTGAAGGAAGACTGTCCTAAGGATTGTAAACTTCTTTTATACGGGAATAACGGGCGATACGAGTATTGCATTGAATGTACCGTAAGAATAAGCATCGGCTAACTCCGTGCCAGCAGCCGCGGTAATACGGAGGATGCGAGCGTTATCCGGATTTATTGGGTTTAAAGGGTGCGTAGGTTGTTCGGTAAGTCAGCGGT | | *Bacteroidetes* | *Bacteroides* | *Bacteroidales* | *Porphyromonadaceae* | *Porphyromonas* |
| *Prevotella loescheii \| HOT-658 \| strain ATCC 15930 \| L16481 \| Named* | TTAGAGTTTGATCATGGCTCAGGATGGACGCTAGCTACAGGCTTAACACATGCAAGTCGCGGGGCAGCATGGGGGTTGCTTGCAACTCCCGATGGCGACCGGCGCACGGGTGAGTAACGCGTATCCAACCTGCCCTTCACCACGGGATAACCCGGCGAAAGTCGGACTAATACCGTATGTTGTCCATTGACGGCATCCGATTTGGACGAAAGGCTTTGCGGTGAAGGATGGGGATGCGTCCGATTAGCCAGACGGCGGGGTAACGGCCCACCGTGGCTACGATCGGTAGGGGTTCTGAGAGGAAGGTCCCCCACACTGGAACTGAGACACGGTCCAGACTCCTACGGGAGGCAGCAGTGAGGAATATTGGTCAATGGGCGTAAGCCTGAACCAGCCAAGTAGCGTGCAGGATGACGGCCCTATGGGTTGTAAACTGCTTTTATGCGGGGATAAAGTGGCCCACGTGTGGGTTTTTGCAGGTACCGCATGAATAAGGACCGGCTAATTCCGTGCCAGCAGCCGCGGTAATACGGAAGGTCCGGGCGTTATCCGGATTTATTGGGTTTAAAGGGAGCGTAGGCCGCGCCTTAAGCGTGTTGT | | *Bacteroidetes* | *Bacteroides* | *Bacteroidales* | *Prevotellaceae* | *Prevotella* |
| *Prevotella melaninogenica \| HOT-469 \| strain ATCC 25845 \| AY323525 \| Named* | TTTACGGTTACCTTGTTACGACTTAGCCCCAATCACCAGTTTTGCCCTAGGCCGATCCTTGCGGT CACGGACTTCAGGCACCCCCGGCTTTCATGGCTTGACGGGCGGTGTGTACNAGGCCCGGGAACNTATTCACCGCGCCATGGCTGATGCNCGATTACTAGCGAATCCGGCNTCGNNGANTNNGNNTGCANANTNTNGTNNAAAANNANGCGGGG | | *Bacteroidetes* | *Bacteroides* | *Bacteroidales* | *Prevotellaceae* | *Prevotella* |
| *Prevotella sp. \| HOT-472 \| clone GU027 \| AY349398 \| Unnamed* | TTAGAGTTTGATCATGGCTCAGGATGGACGCTAGCTACAGGCTTAACACATGCAAGTCGCGGGGCAGCATGGGGGTTGCTTGCAACTCCCGATGGCGACCGGCGCACGGGTGAGTAACGCGTATCCAACCTGCCCTTCACCACGGGATAACCCGGCGAAAGTCGGACTAATACCGTATGTTGTCCATTGACGGCATCCGATTTGGACGAAAGGCTTTGCGGTGAAGGATGGGGATGCGTCCGATTAGCCAGACGGCGGGGTAACGGCCCACCGTGGCTACGATCGGTAGGGGTTCTGAGAGGAAGGTCCCCCACACTGGAACTGAGACACGGTCCAGACTCCTACGGGAGGCAGCAGTGAGGAATATTGGTCAATGGGCGTAAGCCTGAACCAGCCAAGTAGCGTGCAGGATGACGGCCCTATGGGTTGTAAACTGCTTTTATGCGGGGATAAAGTGGCCCACGTGTGGGTTTTTGCAGGTACCGCATGAATAAGGACCGGCTAATTCCGTGCCAGCAGCCGCGGTAATACGGAAGGTCCGGGCGTTATCCGGATTTATTGGGTTTAAAGGGAGCGTAGGCCGCGCCTTAAGCGTGTTGT | | *Bacteroidetes* | *Bacteroides* | *Bacteroidales* | *Prevotellaceae* | *Prevotella* |
| *Propionibacterium acnes \| HOT-530 \| strain 63597 \| AF145256 \| Named* | TTAGAGTTTGATCATGGCTCAGGACGAACGCTGGCGGCGTGCTTAACACATGCAAGTCGAACGGAAAGGCCCTGCTTTTGTGGGGTGCTCGAGTGGCGAACGGGTGAGTAACACGTGAGTAACCTGCCCTTGACTTTGGGATAACTTCAGGAAACTGGGGCTAATACCGGATAGGAGCTCCTGCTGCATGGTGGGGGTTGGAAAGTTTCGGCGGTTGGGGATGGACTCGCGGCTTATCAGCTTGTTGGTGGGGTAGTGGCTTACCAAGGCTTTGACGGGTAGCCGGCCTGAGAGGGTGACCGGCCACATTGGGACTGAGATACGGCCCAGACTCCTACGGGAGGCAGCAGTGGGGAATATTGCACAATGGGCGGAAGCCTGATGCAGCAACGCCGCGTGCGGGATGACGGCCTTCGGGTTGTAAACCGCTTTCGCCTGTGACGAAGCGTGAGTGACG GTAATGGGTAAAGAAGCACCGGCTAACTACGTGCCAGCAGCCGCGGTGATACGTAGGGTGCGAGCGTTGTCCGGATTTATTGGGCGTAAAGGGCTCGTAGGTGGTTGATCGCGTCGGAAGTGTAATCTTGGGGCTTAACCCTG | | *Actinobacteria* | *Actinobacteria* | *Actinomycetales* | *Propionibacteriaceae* | *Propionibacterium* |
| *Pseudomonas fluorescens \| HOT-612 \| strain DSM 50090 \| Z76662 \| Named* | TTAGAGTTTGATCATGGCTCAGATTGAACGCTGGCGGCANGCCTAACACATGCAAGTCGAGCGGTAGAGAGAAGCTTGCTTCTCTTGAGAGCGGCGGACGGGTGAGTAATGCCTAGGAATCTGCCTGGTAGTGGGGGATAACGTTCGGAAACGGACGCTAATACCGCATACGTCCTACGGGAGAAAGCAGGGGACCTTCGGGCCTTGCGCTATCAGATGAGCCTAGGTCGGATTAGCTAGTTGGTGAGGTAATGGCTCACCNAGGCGACGATCCGTAACTGGTCTGAGAGGATGATCAGTCACACTGGAACTGAGAACGGTCCAGACTCCTACGGGAGGCAGCAGTGGGGAATATTGGACAATGGGCGAAAGCCTGATCCAGCCATGCCGCGTGTGTGAAGAAGGTCTTCGGATTGTAAAGCACTTTAAGTTGGGAGGAAGGGCAGTTACCTAATACGTGATTGTTTTGACGTTACCGACAGAATAAGCACCGGCTAACTCTGTGCCAGCAGCCGCGGTAATACAGAGGGNGCAAGCGTTAATCGGAATTACTGGGCGTAAAGCGCGCGTANGTGGTTANNNAANNNNNNTGTGAAATC | | *Proteobacteria* | *Gammaproteobacteria* | *Pseudomonadales* | *Pseudomonadaceae* | *Pseudomonas* |
| *Pseudomonas pseudoalcaligenes \| HOT-740 \| strain LMG 1225 \| Z76666 \| Named* | TTAGAGTTTGATCATGGCTCAGATTGAACGCTGGCGGCAGGCCTAACACATGCAAGTCGAGCGGATGACGGGAGCTTGCTCCTTGATTCAGCGGCGGACGGGTGAGTAATGCCTAGGAATCTGCCTGGTAGTGGGGGATAACGTTCGGAAACGGACGCTAATACCGCATACGTCCTACGGGAGAAAGCAGGGGACCTTCGGGCCTTGCGCTATCAGATGAGCCTAGGTCGGATTAGCTAGTTGGTGAGGTAATGGCTCACCAAGGCGACGATCCGTAACTGGTCTGAGAGGATGATCAGTCACACTGGAACTGAGACACGGTCCAGACTCCTACGGGAGGCAGCAGTGGGGAATATTGGACAATGGGCGAAAGCCTGATCCAGCCATGCCGCGTGTGTGAAGAAGGTCTTCGGATTGTAAAGCACTTTAAGTTGGGAGGAAGGGCAGTTACCTAATACGTGATTGTTTTGACGTTACCGACAGAATAAGCACCGGCTAACTCTGTGCCAGCAGCCGCGGTAATACAGAGGGTGCAAGCGTTAATCGGAATTACTGGGCGTAAAGCGCGCGTAGGTGGTTAGTTAAGTTGGATGTNAAAT | | *Proteobacteria* | *Gammaproteobacteria* | *Pseudomonadales* | *Pseudomonadaceae* | *Pseudomonas* |
| *Pseudomonas stutzeri \| HOT-477 \| strain KC \| AF063219 \| Named* | TTACGGCTACCTTGTTACGACTTCACCCCAGTCATGAATCACACCGTGGTAACCGTCCTCCCGAAGGTTAGACTAGCTACTTCTGGTGCAACCCACTCCCATGGTGTGACGGGCGGTGTGTACAAGGCCTGGGAACGTATTCACCGCGACATTCTGATTCGCGATTACTAGCGATTCCGACTTCACGCAGTCGAGTTGCAGACTGCGATCCGGACTACGATCGGTTTTGTGAGATTAGCTCCACCTCGCGGCTTGGCAACCCTCTGTACCGACCATTGTAGCACGTGTGTAGCCCAGGCCGTAAGGGCCATGATGACTTGACGTCATCCCCACCTTCCTCCGGTTTGTCACCGGCAGTCCCTTAGAGTGCCCACCATAACGTGCTGGTAACTAAGGACAAGGGTTGCGCTCGTTACGGGACTTAACCCAACATCTCACGACACGAGCTGACGACAGCCATGCAGCACCTGTGTCAGAGTTCCCGAAGGCACCAATCCATCTCTGGAAAGTTCTCTGCATGTCAAGGCCTGGTAAGGTTCTTCGCGTTGCTTCGAATTAAACCACATGCTCCACCGCTTGTGCGGGCCCCCGTCAATTCA | | *Proteobacteria* | *Gammaproteobacteria* | *Pseudomonadales* | *Pseudomonadaceae* | *Pseudomonas* |
| *Ralstonia pickettii \| HOT-854 \| strain ATCC 27511 \| NR_043152 \| Named* | TTTACGGCTACCTTGTTACGACTTCACCCCAGTCATGAACCCTACCGTGGTAATCGCCCTCCTTGCGGTTAGGCTAACTACTTCTGGTAAAGCCCACTCCCATGGTGTGACGGGCGGTGTGTACAAGACCCGGGAACGTATTCACCGCGGCATGCTGATCCGCGATTACTAGCGATTCCAGCTTCACGTAGTCGAGTTGCAGACTACGATCCGGACTACGATGCATTTTCTGGGATTAGCTCCACCTCGCGGCTTGGCAACCCTCTGTATGCACCATTGTATGACGTGTGAAGCCCTACCCATAAGGGCCATGAGGACTTGACGTCATCCCCACCTTCCTCCGGNTTGNCNCCNGGCAGTCTCTCTAGAGTGCCCTTTCGTAGCAACTAGAGACAAGGGTTGCGCTCGTTGCGGGACTTAA CCCAACATCTCACGACACGAGCTGACGACAGCCATGCAGCACCTGTGTCCACTTTCTCTTTCGAGCACCTAATGCATCTCTGCTTCGTTAGTGGCATGTCAAGGGTAGGTAAGGTTTTTCGCGTTGCATCGAATTAATCCACATCATCCACCGCTTGTGCGGGTCCCCGTCAATTCCTT | | *Proteobacteria* | *Betaproteobacteria* | *Burkholderiales* | *Ralstoniaceae* | *Ralstonia* |
| *Ralstonia sp. \| HOT-027 \| strain C37KA \| AY005039 \| Unnamed* | TTAGAGTTTGATCATGGCTCAGATTGAACGCTGGCGGCATGCCTTACACATGCAAGTCGAAC GGCAGCACGGACTTCGGTCTGGTGGCGAGTGGCGAACGGGGAGTAATATATCGGAACATACCCAGTAGTGGGGGATAACTATCCGAAAGGATAGCTAATACCGCATACGATCTGTGGATGAAAGCAGGGGATCTTCGGACCTTGTGCTATTGGAATGGCCGATATCTGATTAGCTAGTTGGTAGGGTAAAAGCCTACCAAGGCGACGATCAGTAGCTGGTCTGAGAGGACGACCAGCCACACTGGAACTGAGACACGGTCCAGACTCCTACGGGAGGCAGCAGTGGGGAATTTTGGACAATGGGGGCAACCCTGATCCAGCAATGCCGCGTGAGTGAAGAAGGCCCTCGGGTTGTAAAGCTCTTTTGTCAGGGAAGAAACGGAGTTCTCTAATATAGGATTCTAATGACGGTACCTGAAGAATAAGCACCGGCTAACTACGTGCCAGCAGCCGCGGTAAACGTAGGGTGCAAGCGTTAATCGGAATTACTGGGCGTAAAGCGTGCGCAGGCGGTTTTGTAAGCTGATGTGAAATC | | *Proteobacteria* | *Betaproteobacteria* | *Burkholderiales* | *Ralstoniaceae* | *Ralstonia* |
| *Sphingomonas sp. \| HOT-006 \| clone FI012 \| AY349411 \| Phylotype* | TTTACGGTTACCTTGTTACGACTTCACCCCAGTCGCTAAACCCACTGTGGTCGCCTGCCTCCTTGCGGTTAGCTCAACGCCTTCGAGTGAATCCAACTCCCATGGTGTGACGGGCGGTGTGTACAAGGCCTGGGAACGTATTCACCGCGGCATGCTGATCCGCGATTACTAGCGATTCCGCCTTCACGCTCTCGAGTTGCAGAGAACGATCCGAACTGAGACGACTTTTGGAGATTAGCTCCCTCTCGCGAGGTGGCTGCCCACTGTAGTCGCCATTGTAGCACGTGTGTAGCCCAACGCGTAAGGGCCATGAGGACTTGACGTCATCCCCACCTTCCTCCGGCTTATCACCGGCGGTTCCTTTAGAGTACCCAACTAAATATGGCAACTAAAGGCGAGGGTTGCGCTCGTTGCGGGACTTAACCCAACATCTCACGACACGAGCTGACGACAGCCATGCAGCACCTGTCACCTATCCAGCCGAACTGAAGGAAAGTGTCTCCACGATCCGCGATAGGGATGTCAAACGTTGGTAAGGTTCTGCGCGTTGCTTCGAATTAAACCACATGCTCCACCGCTTGTGCAGGCCCCCTCAATT | | *Proteobacteria* | *Alphaproteobacteria* | *Sphingomonadales* | *Sphingomonadaceae* | *Sphingomonas* |
| *Stenotrophomonas maltophilia \| HOT-663 \| strain LMG 958 \| X95923 \| Named* | TTAGAGTTTGATCCTGGCTCAGAGTGAACGCTGGCGGTAGGCCTAACACATGCAAGTCGAACGGCAGCACAGGAGAGCTTGCTCTCTGGGTGGCGAGTGGCGGACGGGTGAGGAATACATCGGAATCTACCTTTTCGTGGGGGATAACGTAGGGAAACTTACGCTAATACCGCATACGACCTACGGGTGAAAGTGGGGGACCGCAAGGCCTCACGCGATTAGATGAGCCGATGTCCGATTAGCTAGTTGGCGGGGTAATGGCCCACCAAGGCGACGATCGGTAGCTGGTCTGAGAGGATGATCAGCCACACTGGAACTGAGACACGGTCCAGACTCCTACGGGAGGCAGCAGTGGGGAATATTGGACAATGGGCGCAAGCCTGATCCAGCCATACCGCGTGGGTGAAGAAGGCCTTCGGGTTGTAAAGCCCTTTTGTTGGGAAAGAAATCCTGTCGGTTAATAACCGGTGGGGATGACGGTACCCAAAGAATAAGCACCGGCTAACTTCGTGCCAGCAGCCGCGGTAATACGAAGGGTGCAAGCGTTACTCGGAATTAC TGGGCGTAAAGCGTGCGTAGGTGGTGGTTNAAGTCTGCTGT | | *Proteobacteria* | *Gammaproteobacteria* | *Xanthomonadales* | *Xanthomonadaceae* | *Stenotrophomonas* |
| *Streptococcus sinensis \| HOT-767 \| strain HKU4 \| AF432856 \| Named* | TTAGAGTTTGATCCTGGCTCAGGACGAACGCTGGCGGCGTGCCTAATACATGCAAGTGGAACGCACGAGGTACACCGTAGT TTACTACATCGTACTTTGTGAGTCGCGAACGGGTGAGTAACGCGTAGGTAACCTACCTTTTAGGGGGGATAACTATTGGAAACGATAGCTAATACCGCATAAGATATCTTACTGCATGGTAAGATGTTAAAAGATGCAATTGCATCACTAAGAGATGGACCTGCGTTGTATTAGCTAGTAGGTGAGGTAACGGCTCACCTAGGCAACGATACATAGCCGACCTGAGAGGGTGATCGGCCACACTGGGACTGAGACACGGCCCAGACTCCTACGGGAGGCAGCAGTAGGGAATCTTCGGCAATGGGGGGAACCCTGACCGAGCAACGCCGCGTGAGTGAAGAAGGTTTTCGGATCGTAAAGCTCTGTTGTAAGAGAAGAACGAGTGTGAGAGTGGAAAT TCACACTGTGACGGTAACTTACCAGAAAGGGACGGCTAACTACGTGCCAGCAGCCGCGGTAATACGTAGGTCCCGAGCGTTGTCCGGATTTATTGGGCGTAAAGCGAGCGCAGGCGGTT | | *Firmicutes* | *Bacilli* | *Lactobacillales* | *Streptococcaceae* | *Streptococcus* |
| *Streptococcus sp. \| HOT-064 \| clone C5MLM037 \| AY278609 \| Phylotype* | TTAGAGTTTGATCATGGCTCAGGACGAACGCTGGCGGCGTGCCTAATACATGCAAGTAGAACGCTGAAGGAGGAGCTTGCTCTTCTGGATGAGTTGCGAACGGTGAGTAACGCGTAGGTAACCTGCCTGGTAGCGGGGGATAACTATTGGAAACGATAGCTAATACCGCATAAGAGTAGATGTTGCATGACATTTGCTTAAAAGGTGCAATTGCATCACTACCAGATGGCCTGCGTTGTATTAGCTAGTTGGTGAGGTAACGGCTCACCAAGGCAACGATACATAGCCGACCTGAGAGGGTGATCGGCCACACTGGGACTGAGACACGGCCCAGACTCCTACGGGAGGCAGCAGTAGGGAATCTTCGGCAATGGACGGAAGTCTGACCGAGCAACGCCGCGTGAGTGAAGAAGGTTTTCGGATCGTAAAGCTCTGTTGTAAGAGAAGAACGAGTGTGAGAGTGGAAAGTTCACACTGTGACGGTATCTTACCAGAAAGGGACGGCTAACTACGTGCCAGCACCGCGGTAATACGTAGGTCCCGAGCGTTGTCCGGATTTATTGGGCGTAAAGCGAGCGCAGGCGGTTAGATAAGTCT | | *Firmicutes* | *Bacilli* | *Lactobacillales* | *Streptococcaceae* | *Streptococcus* |
| *Treponema vincentii \| HOT-029 \| strain ATCC 35580 \| AF033309 \| Named* | TTTACGGCTACCTTGTTACGACTTCACCCTCCTTACCAAGCGTCCTTCGGCACCGTCCTCCTTTGCAGGTTAGACAAGCGACTTCGGGTACCCCCAACTCGGATGGTGTGACGGGCGGTGTGTACAAGGCCCGGGAACGTATTCACCNNNNCCGNGCTGATGCGCGATTACTAGCGATTCCAACTTCATGAAGTNNAAGTTTCAGACTTCAATCCGAACTACGATTGCTTTTTTGCGGTTTGCTCCACCTTACGGTCTTGCTTCGCTTTGTAGCAACCATTGTAGCACGTGTGTAGCCCT GGACATAAGGGGCATGATGACTTGACGTCATCCCCGCCTTCTCCGCGTTCTCCGCGGCAGTCTCCTTTGAGTGCCCAGCTTTACCTGATGGCAACAAAGAACAGGGGTTGCGCTCGTTGCGGGACTTAACCCAACATCTCACGACACGAGCTGACGACAACCATGCACACCTGTCTCCTCTGTCCGAAGAAATACCCGATTAAGGGTATGTCAGAGGGATGTCAAGTCTTGTAAGGTTCTTCGCGTTGCTTCGAATTAAACCACATGCTCCGCTGCTTGTGCGGGTCCCCGTCAT | | *Spirochaetes* | *Spirochaetes* | *Spirochaetales* | *Spirochaetaceae* | *Treponema* |
| *Veillonella atypica \| HOT-524 \| strain DSM 20739 \| X84007 \| Named* | CAATCATCGACTTTACCTTAGACGGCTGGCTCCCGAAGGTTACCCCACCGGCTTTGGGCACTTCCGACTTTCGTGGTGTGACGGGCGGTGTGTACAAGGCCCGGGAACGTATTCACCGCAGTATGCTGACCTGCGATTACTAGCGATTCCGACTTCACGTAGGCGAGTTGCAGCCTACGATCCGAACTGAGAGAGTGTTTCTCGGGTTTGCTCCACCTCGCGGTATTGCTTCCGTCTATTAACTCCCATTGTAGTACGTGTGTAGCCCAGGTCATAAGGGGCATGATGATTTGACGTCATCCCCGCCTTCCTCCGCATTGTCTGCGGCAGTCTCTCATGAGTTCCCACCCGAAGTGCTGCAACATAAGATAGGGGTTGCGCTCGTTGCGGGACTTAACCCAACATCTCACGACACGAGCTGACGACAACCGTGCACCACCTGTTTCTGGCTTCCGAAGAAGAGGAACCATCTCTGGTTCTGTCCATCAATGTCAAGACCTGGTAAGGTTCTTCGCGTTGCGTCGAATTAACCACATACTCCACCGCTTGTGCGGGCCCCCGTCAATTCCT | | *Firmicutes* | *Clostridia* | *Clostridiales* | *Veillonellaceae* | *Veillonella* |
| *Veillonella parvula \| HOT-161 \| clone BU083 \| AF366266 \| Named* | TTCGGCTACCTTGTTACGACTTCACCCCAATCATCGACTTTACCTTAGACGGCTGGCTCCCGAAGTTACCCCACCGGCTTTGGGCACTTCCGACTTTCGTGGTGTGAGGGCGGTGTGTACAAGGCCCGGGAACGTATTCACCGCAGTATGCTGACCTGCGATTACTAGCGATTCCGACTTCACGTAGGCGAGTTGCAGCCTACGATCCGAACTGAGAGAGTGTTTCTCGGGTTTGCTCCATCTCGCGATCTCGCTTCCGTCTATTAACTCCCATTGTAGTACGTGTGTAGCCCAGGTCATAAGGGGCATGATGATTTGACGTCATCCCCGCCTTCCTCCGCATTGTCTGCGGCAGTCTCTCATGAGTTCCCACCATTACGTGCTGGCAACATAAGATAGGGGTTGCGCTCGTTGCGGGACTTAAC CCAACATCTCACGACACGAGCTGACGACAACCGTGCACCACTGTTTTCTGGCTTCCGAAGAAGAGGAACTATCTCTAGTTCTGTCCATCAATGTCAAGACCTGGTAAGGTTCTTCGCGTTGCGTCGAATTAAACCACATACTCCACCGCTTGTGCGGGCCCCCGTCAATT | | *Firmicutes* | *Clostridia* | *Clostridiales* | *Veillonellaceae* | *Veillonella* |
| *Veillonella rogosae \| HOT-158 \| clone BI029 \| GQ422725 \| Named* | TTAGAGTTTGATCCTGGCTCAGGACGAACGCTGGCGGCGTGCTTAACACATGCAAGTCGAAGAAGAGCGATGGAAGCTTGCTTCTATCAATCTTAGTGGCGAACGGGTGAGTAACGCGTAATC AACCTGCCCTTCAGAGGGGGACAACAGTTGGAAACGACTGCAATACCGCATACGATCCAATCTCGGCATCGAGGATGGATGAAAGGTGGCCTCTATTTATAAGCTATCACTGAAGGAGGGGATTGCGTCTGATTAGCTAGTTGGAGGGGTAACGGCCCACCAAGGCAATGATCAGTAGCCGGTCTGAGAGGATGAACGGCCACATTGGGACTGAGACACGGCCCAGACTCCTACGGGAGGCAGCAGTGGGGAATCTTCCGCAATGGACGAAAGTCTGACGGAGCAACGCCGCGTGAGTGATGACGGCCTTCGGGTTGTAAAGCTCTGTTAATCGGGACGAAAGGTCCTCTTGCGAATAGTTAGAGGAATTGACGGTACCGGAATAGAAAGCCACGGCTAACTACGTGCCAGCAGCCGCGGTAATACGTAGGTGGCAAGCGTTGTCCGGAATTATTGGGGTAAAGCGCGCGCAGG | | *Firmicutes* | *Clostridia* | *Clostridiales* | *Veillonellaceae* | *Veillonella* |
